# Seasonal Dynamics of Phlebotomine Sand Fly Species Proven Vectors of Mediterranean Leishmaniasis Caused by *Leishmania infantum*

**DOI:** 10.1371/journal.pntd.0004458

**Published:** 2016-02-22

**Authors:** Bulent Alten, Carla Maia, Maria Odete Afonso, Lenea Campino, Maribel Jiménez, Estela González, Ricardo Molina, Anne Laure Bañuls, Jorian Prudhomme, Baptiste Vergnes, Celine Toty, Cécile Cassan, Nil Rahola, Magali Thierry, Denis Sereno, Gioia Bongiorno, Riccardo Bianchi, Cristina Khoury, Nikolaos Tsirigotakis, Emmanouil Dokianakis, Maria Antoniou, Vasiliki Christodoulou, Apostolos Mazeris, Mehmet Karakus, Yusuf Ozbel, Suha K. Arserim, Ozge Erisoz Kasap, Filiz Gunay, Gizem Oguz, Sinan Kaynas, Nikoloz Tsertsvadze, Lamzira Tskhvaradze, Ekaterina Giorgobiani, Marina Gramiccia, Petr Volf, Luigi Gradoni

**Affiliations:** 1 Department of Biology, Ecology Division, HUESRL-VERG laboratories, Hacettepe University, Ankara, Turkey; 2 Instituto de Higiene e Medicina Tropical, Unidade Parasitologia Médica, Universidade Nova de Lisboa, Portugal; 3 Centro Nacional de Microbiologia, Servicio de Parasitología, Unidad de Entomología Médica, Instituto de Salud Carlos III, Majadahonda, Madrid, Spain; 4 Institut de Recherche pour le Développement, MIVEGEC, Montpellier, France; 5 M.I.P.I. Department, Unit of Vector-borne Diseases and International Health, Istituto Superiore di Sanità, Rome, Italy; 6 Laboratory of Clinical Bacteriology, Parasitology, Zoonoses and Geographical Medicine, University of Crete, Heraklion, Greece; 7 Veterinary Services of Cyprus, Nicosia, Cyprus; 8 Department of Parasitology, Ege University, Izmir, Turkey; 9 Vocational School of Health Sciences, Cela Bayar University, Manisa, Turkey; 10 Veterinary Faculty, Mehmet Akif Ersoy University, Burdur, Turkey; 11 National Center for Disease Control and Public Health, Tbilisi, Georgia; 12 Department of Parasitology, Faculty of Science, Charles University, Prague, Czech Republic; Lancaster University, UNITED KINGDOM

## Abstract

**Background:**

The recent geographical expansion of phlebotomine vectors of *Leishmania infantum* in the Mediterranean subregion has been attributed to ongoing climate changes. At these latitudes, the activity of sand flies is typically seasonal; because seasonal phenomena are also sensitive to general variations in climate, current phenological data sets can provide a baseline for continuing investigations on sand fly population dynamics that may impact on future scenarios of leishmaniasis transmission. With this aim, in 2011–2013 a consortium of partners from eight Mediterranean countries carried out entomological investigations in sites where *L*. *infantum* transmission was recently reported.

**Methods/Principal Findings:**

A common protocol for sand fly collection included monthly captures by CDC light traps, complemented by sticky traps in most of the sites. Collections were replicated for more than one season in order to reduce the effects of local weather events. In each site, the trapping effort was left unchanged throughout the survey to legitimate inter-seasonal comparisons. Data from 99,000 collected specimens were analyzed, resulting in the description of seasonal dynamics of 56,000 sand flies belonging to *L*. *infantum* vector species throughout a wide geographical area, namely *P*. *perniciosus* (Portugal, Spain and Italy), *P*. *ariasi* (France), *P*. *neglectus* (Greece), *P*. *tobbi* (Cyprus and Turkey), *P*. *balcanicus* and *P*. *kandelakii* (Georgia). Time of sand fly appearance/disappearance in collections differed between sites, and seasonal densities showed variations in each site. Significant correlations were found between latitude/mean annual temperature of sites and i) the first month of sand fly appearance, that ranged from early April to the first half of June; ii) the type of density trend, varying from a single peak in July/August to multiple peaks increasing in magnitude from May through September. A 3-modal trend, recorded for *P*. *tobbi* in Cyprus, represents a novel finding for a *L*. *infantum* vector. Adults ended the activity starting from mid September through November, without significant correlation with latitude/mean annual temperature of sites. The period of potential exposure to *L*.*infantum* in the Mediterranean subregion, as inferred by adult densities calculated from 3 years, 37 sites and 6 competent vector species, was associated to a regular bell-shaped density curve having a wide peak center encompassing the July-September period, and falling between early May to late October for more than 99% of values. Apparently no risk for leishmaniasis transmission took place from December through March in the years considered. We found a common pattern of nocturnal females activity, whose density peaked between 11 pm and 2 am.

**Conclusions:**

Despite annual variations, multiple collections performed over consecutive years provided homogeneous patterns of the potential behavior of leishmaniasis vectors in selected sites, which we propose may represent sentinel areas for future monitoring. In the investigated years, higher potential risk for *L*. *infantum* transmission in the Mediterranean was identified in the June-October period (97% relative vector density), however such risk was not equally distributed throughout the region, since density waves of adults occurred earlier and were more frequent in southern territories.

## Author Summary

Recent projections on global warming indicate a constant rise of temperatures in the Mediterranean subregion in the near-mid future. While this phenomenon already caused geographical expansion of several arthropod-borne diseases, it is likely to affect also temporal parameters of seasonally transmitted diseases such as leishmaniasis, a protozoan infection spread by the bite of phlebotomine sand flies. Phenology of sand flies consists in the periods of emergence of adults and their disappearance from collections following an activity period during warm months, which can be characterized by peaks of abundance. Current phenological observations can be important for continuing investigations on sand fly dynamics that may impact on leishmaniasis transmission in the future. With this aim, partners from eight Mediterranean countries identified sites with documented *Leishmania infantum* transmission by six different vector species and performed multiannual trappings. From the high number of 56,000 specimens collected throughout an area spanning from Portugal at west to Georgia at east, the current seasonal dynamics of Mediterranean vectors was obtained. Both, period of adults emergence and type of density trend were found to be significantly correlated with latitude or mean annual temperature of trapping sites. At the southernmost latitudes, vector activity started as early as begin of April and ended by late November, showing that the no-risk period of potential exposure to *L*. *infantum* lasted only 4 months.

## Introduction

Phlebotomine sand flies (Diptera, Psychodidae) are the unique haematophagous insects proven to transmit leishmaniases [1]. Of approximately 900 species estimated to exist [2] less than a hundred, belonging to *Phlebotomus* and *Lutzomyia* (*sensu* Young and Duncan, 1994 [3]) genera are proven or suspected vectors of human disease in the Old and New Worlds, respectively [4,5]. *Leishmania infantum* is the main causative agent of zoonotic visceral leishmaniasis (VL) and also responsible for cases of cutaneous leishmaniasis (CL) accross the Mediterranean subregion that includes southern Europe, northern Africa and parts of Asia [6,7]. Domestic dogs are primary reservoirs of infection for humans and may suffer from a severe chronic disease (canine leishmaniasis, CanL). The annual incidence of human VL is estimated to range from 1500 to 2700 cases; available figures on CL caused by *L*. *infantum* are low reliable because of poor reporting [8]. A dozen of *Phlebotomus* species have been implicated in the transmission of Mediterranean *L*. *infantum*, of which eight have been incriminated as vectors according to conventional criteria [4,9]: *Phlebotomus ariasi* [10], *P*. *balcanicus* [11], *P*. *kandelakii* [11], *P*. *langeroni* [12], *P*. *neglectus* [13], *P*. *perfiliewi* [14], *P*. *perniciosus* [15] and *P*. *tobbi* [16]. All these species but *P*. *balcanicus*, which belongs to the subgenus *Adlerius*, are members of the *Larroussius* subgenus.

Effects of long-term climate changes on the geographical expansion of Mediterranean *L*. *infantum* vectors towards northern latitudes or higher altitudes have been predicted [17] and actually observed in some places, such as in northern Italy [18, 19], French and Spanish Pyrenees [20,21] and in Rhineland-Palatinate, Germany [22]. As a consequence, spread of human and canine disease associated with progressive increase of *Leishmania* seroprevalence rates in dogs were reported at altitudes and latitudes higher than might be expected in several Mediterranean countries [reviewed in [23]]. The activity period of Mediterranean adult sand flies is typically seasonal. Because seasonal phenomena are also very sensitive to variations in temperature, phenological observations may provide high resolution of ongoing climate changes in addition to geographical dispersion parameters [24]. However the available information on sand fly seasonal dynamics in the Mediterranean region is patchy, being disperse in time and space. Although several investigations on this subject were performed over the past 50 years, they included mostly one season—rarely 2 or 3—and were performed in single sites at different periods [e.g. 11, 25–31]. Hence, recent and accurate knowledge of Mediterranean vectors dynamics would be required as a starting baseline for continuing investigations on changes that may have an impact on leishmaniasis transmission. Because investigations of this type are prone to several confounding parameters, prerequisites would be: a) to perform studies in a relatively short period (i.e. a time range of a few years) in order to minimize possible effects of the ongoing slow temperature increase [32]; b) to replicate studies in the same sites for more than one season, with a view to reduce as far as possible the effects of annual climate variations and local weather events; and c) to carry out studies over a wide geographical area, in order to provide general information from different latitudes and longitudes.

In the frame of EDENext EU FP7 (www.edenext.eu) project, in 2011–2014 a consortium of partners from 8 Mediterranean countries endemic for *L*. *infantum* have carried out entomological investigations in representative sites for at least 2 consecutive years. In this paper, we report data from about 99000 collected specimens, resulting in the description of seasonal dynamics of about 56000 sand flies belonging to species competent to transmit *L*. *infantum* over a wide geographical range spanning from Portugal at west to Georgia at east, namely: *P*. *perniciosus* (Portugal, Spain and Italy); *P*. *ariasi* (France); *P*. *neglectus* (Greece); *P*. *tobbi* (Cyprus and Turkey); *P*. *balcanicus* and *P*. *kandelakii* (Georgia).

## Materials and Methods

### Study areas and vectors

**Fig 1:**
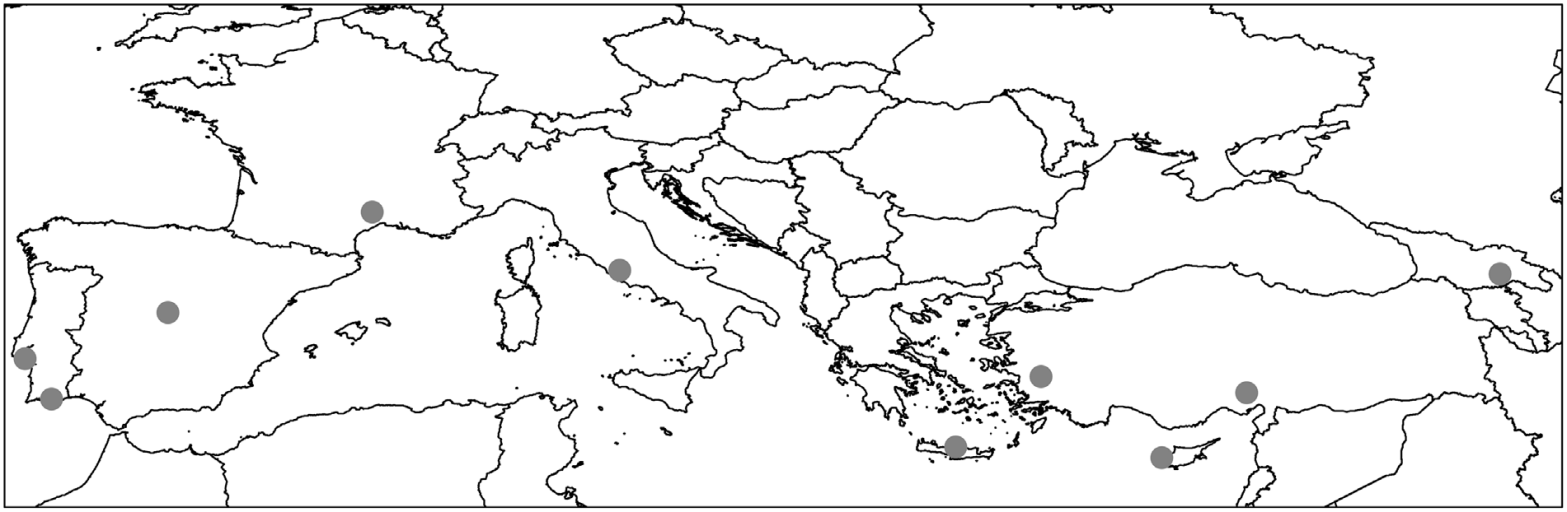
Location of the sand fly collecting sites in the Mediterranean region.

In each country, one or more locations were identified on the basis of the historical presence of proven phlebotomine vector(s) and the evidence of human and/or animal leishmaniasis transmission in the area. Geographical coordinates and altitude of study regions and sites are shown in Table 1. The southernmost site was located in Cyprus (latitude 34°59’54”N) and the northernmost in Languedoc-Roussillon, France (latitude 43°58’23”N). The westernmost site was in Setúbal district, Portugal (longitude 9°16’52”W) and the easternmost in Tbilisi, Georgia (longitude 44°49’30”E). All sites were at low-mid altitudes above sea level: the lowest altitude was in Portugal (3 m), the highest in Spain (691 m). On overview of the collecting sites location in the Mediterranean region is shown in Fig 1.

**Table 1:** Geographical coordinates and elevation of 37 sand fly collecting sites.

Country	Region/District/Site*	No. of sites	Latitude	Longitude	Elevation (m a.s.l.)
Portugal	Lisbon Metropolitan Region/Setúbal and Lisbon	11	From 38°28’37”N to 38°44’51”N	From 9°16’52”W to 8°45’2”W	3–330
	Algarve/Faro	11	From 37°3’27”N to 37°14’20”N	From 8°37’45”W to 7°26’34”W	10–74
Spain	Autonomous Community of Madrid/Fuenlabrada	1	40°17’53”N	3°47’31”W	635–691
France	Languedoc-Roussillon/ Gard/Roquedur-le-haut	1	43°58’23”N	3°39’26”E	603
Italy	Latium/Rome/Frascati	1	41°50’34”N	12°41'57”E	192
Greece	Crete/Heraklion/Fodele	1	35°22’52”N	24°57'29”E	40–70
Cyprus	Paphos/Steni	1	34°59’54” N	32°28'17” E	200
Turkey	Aegean/Aydin/Bascayir	1	37°57’35”N	28°04’03”E	427
	Cukurova/Adana	6	From 37°17’59”N to 37°26’01”N	From 35° 31’01”E to 35°39’27”E	150–280
Georgia	Tbilisi/Gldani-Nadzaladevi and Isani-Samgori	3	From 41°42’01”N to 41°44’08”N	From 44° 48’59”E to 44°49’30”E	495–603

*Named when only one site was investigated

In Portugal, the Lisbon Metropolitan Region in the centre and the Algarve Region in the south are endemic for VL, CL and CanL, predominantly caused by the *L*. *infantum* zymodeme MON-1 and transmitted by two vectors, *P*. *perniciosus* and *P*. *ariasi* [33,34]. The investigations required collections in multiple sites of the two Regions, because of the known low density of local vectors in most places. Thus, 11 collecting sites from the Setúbal and Lisbon Departments in Lisbon Metropolitan Region were investigated in 2011 and 2012; 11 sites from the Department of Faro in Algarve Region were investigated in 2011, 2012 and 2013.

In Spain, an outbreak of both VL and CL has occurred since mid 2009 in four towns southwest of Madrid, with nearly 600 cases mainly recorded in Fuenlabrada [35]. In contrast to a low prevalence of CanL, lagomorphs—the hare *Lepus granatensis* and the wild rabbit *Oryctolagus cuniculus*—were identified as unusual reservoir in a large urban park. These animals have been found infected by *L*. *infantum* at high rates and proved to transmit the parasite to reared *P*. *perniciosus* [36]. Wild *P*. *perniciosus*, the only proven vector in the area, was found to harbor the same strain of *L*. *infantum* causing human and animal infections [37]. Locations bordering the urban center of Fuenlabrada were surveyed in this endemic setting in 2012 and 2013.

In southern France, a traditional endemic focus of human leishmaniasis and CanL caused by *L*. *infantum* is represented by a hilly area located between the Hérault and Arre valleys in the Languedoc-Roussillon Region. The dominant phlebotomine species in the area is *P*. *ariasi*, incriminated as *L*. *infantum* vector in late 1970s [10]. A representative site (Roquedur-le-haut) was investigated in 2011, 2012 and 2013.

In Italy, the central Latium Region is ranked as the third most endemic territory for human VL and CanL, both caused by *L*. *infantum* MON-1 [38]. *P*. *perniciosus* is the proven vector in Rome province [29] and this species is found abundant in hills surrounding the capital city. A site from this area (Frascati) was surveyed in 2011 and 2012.

In Greek islands and mainland, leishmaniases have re-emerged during the past three decades [39]. In Crete, both *L*. *infantum* zymodemes MON-1 and MON-98 were found to cause human VL and CanL. Among 9 *Phlebotomus* species endemic in the island, *P*. *neglectus*, a proven *L*. *infantum* vector in Greece [13], is the most abundant and geographically widespread [40]. A representative site (Fodele, Heraklion region) was investigated from 2011 through 2013.

In Cyprus, leishmaniasis has re-emerged in recent years; apparently no human cases caused by *L*. *infantum* occur in the island, despite this parasite (predominantly zymodeme MON-1) is widespread among dogs [41]. *P*. *tobbi* is the proven *L*. *infantum* vector in Cyprus [16], whereas the role of this species in the transmission of the recently introduced *Leishmania donovani* MON-37 (apparently the only cause of human VL and CL in the island [23]) remains to be determined. A representative collection site (Steni, Paphos district) was investigated in 2012 and 2013.

Different entities of human leishmaniasis are endemic in Turkey, including zoonotic VL and CL caused by *L*. *infantum*, and anthroponotic CL by *L*. *tropica* [42]. *L*. *infantum* was traditionally prevalent in the Aegean and Mediterranean coasts of western Turkey, however it has been recently detected as an agent of CL in Cukurova region, south Anatolia [43]; genome sequencing of strains isolated from *P*. *tobbi* and human patients revealed *L*. *infantum*/ *L*. *donovani* hybrids [44]. Among at least 21 *Phlebotomus* species described in Turkey, a few potential *L*. *infantum* vectors have been suspected throughout the country, but only *P*. *tobbi* has been incriminated conclusively [43]. Six collecting sites were selected in villages of the northwest part of the Cukurova region, Adana province, for seasonal dynamics investigations in 2011 and 2012. In western Turkey, a representative site (Bascayr village, Aydin province) endemic for human VL and CanL and showing predominance of the vector *P*. *tobbi* [45], was selected for investigations on nocturnal activity in 2012.

In Georgia, the disease has emerged as a significant public threat in the capital city of Tbilisi beginning from the years 1990s. The infections, caused by *L*. *infantum*, were also found widespread in domestic and stray dogs [46]. In this endemic setting, 2 phlebotomine species have been recently incriminated as *L*. *infantum* vectors, *P*. *kandelakii* and *P*. *balcanicus* [11]. Two collecting sites were selected for phenological investigations performed from 2011 through 2013 in each of the two urban districts where VL mostly occurs: Lotkini in Gldani-Nadzaladevi district, and Elia in Isani-Samgori district, respectively. Investigations on nocturnal activity were performed in 2014 in the Vera area of Tbilisi.

### Sand fly collection, habitat and species identification

A common protocol for trapping methods and periodicity of sand fly collections was established. Priority was given to the use of CDC miniature light traps equipped with a fine net cage, which were used by all investigators and whose number in each site had to remain unchanged over the years to legitimate comparisons between seasons. One or the other of two common models, considered equivalent for the study purpose (Hausherr’s Machine Works, Toms River, New Jersey, USA; John W. Hock Co., Gainesville, Florida, USA) were placed in each collection site for at least 2 consecutive nights per month, with replacement of traps after every night. Most of the teams, however, set light traps for 2 nights or more every 15 days. Previous investigator’s experience in each setting showed that trapping yields may vary greatly depending whether sand flies concentrate in hot spots, thus requiring the use of a few traps, or they are dispersed at low density over a territory, which requires a greater sampling effort. Thus, the number of light traps varied from 3 to 15 depending on site, for a total sampling effort ranging from 56 (in 2013 surveys, 6 countries) to 94 traps (in 2012 surveys, 8 countries) per night of capture. They were set operating 1–2 hours before sunset until 1–2 hours after sunrise. For specific investigations on the nocturnal activity of vectors, light traps operated for at least one night in a selected site, and manual or automatic trap replacement was performed every 1 or 2 hours. Temperature and humidity were recorded daily during the trapping period.

Light-attraction collections were complemented by the use of sticky traps in most of the surveyed sites, the choice depending on previous investigator’s experience on sand fly yields by both trapping methods. For example, in sites of Cukurova region and Tbilisi interception methods were found to be low productive so that only light traps were employed there. Sticky traps consisted of 20x20 cm white paper coated with castor oil; again, a fixed number of sticky traps per site was set monthly for at least 2 consecutive days.

The start of trapping activity was based on previous local experience. It was established that investigators had to bring collections forward by one month compared to historical observations on the local sand fly appearance. Trapping was stopped when no more sand flies were captured by any method. Because it was considered virtually impossible that teams from different countries could perform simultaneously sand fly trapping exactly at the same annual days, the common protocol established that, within each month, setting of traps had to be made in a period having day 15 as median. Each team maintained a fixed periodicity of collections in the surveyed territory and, among all teams, catches were actually performed over a maximum range of ± 12 days from the median with a standard deviation of 8 days.

A large variety of domestic and peri-domestic, rarely sylvatic, habitats were surveyed in rural, village or urban areas by means of indoor and/or outdoor collections. In general, they were fairly representative of typical *L*. *infantum* transmission biotopes in the Mediterranean region. Traps were set in human dwellings, cellars, courtyards, livestock sheds, hen houses, kennels, gardens, shady areas in various types of orchards, olive groves, and vineyards. The large diversity of potential blood meal sources included humans and domestic animals such as dogs, cats, cattle, sheep, goats, horses, rabbits, and poultry. A number of wild mammals of potential interest in *L*. *infantum* epidemiology were also present in surrounding areas (e.g. black rats, hares, wild rabbits and foxes).

Collected sand flies were preserved in ethanol pending species identification. This was largely performed morphologically using published keys and descriptions [47–52]. Specimen identification of the phlebotomine fauna belonging to taxonomically challenging species (e.g. *P*. *transcaucasicus*/*P*. *galilaeus* or *P*. *sergenti/P*. *similis*) was also confirmed by morphometric measurements [53] and, for some representative specimens, by sequencing analysis of two gene markers, namely cytochrome b [54] and internal transcribed spacer 2 (ITS2) [55]. As regards female members of the *Adlerius* subgenus, for which full taxonomic keys are not available, they were identified with associated males and by differences in pharyngeal armature following Artemiev’s drawings [50].

### Data analysis and output

The final outcome of this study was the production in practical graphic format of phenological and abundance parameters that may have an impact on *L*. *infantum* transmission by relevant vector species in each endemic settings and the whole subregion, namely: a) the beginning and the end of adults activity; b) the variation in abundance during the activity period; c) the nocturnal activity. Starting from large databases produced for each site, various data levels were combined *a priori* (e.g. in case of collections performed in multiple sites within a small territory) or in course of data analysis. For example, specimen densities from both sexes were combined in the description of vector population dynamics in single or multiple seasons, whereas only female densities were considered in the evaluation of nocturnal activity. In addition to annual data, the potential behavior of vectors in each endemic setting was evaluated considering combined data from all years of collection. Monthly densities expressed as number of vector specimens/light trap were presented as such in most of the graphs. Correlation analyses were generated by Pearson correlation coefficient; statistics and graph production were performed using GraphPad Prism Software, version 5.00 for Windows (San Diego, California, USA).

## Results

### Sand fly fauna

A total of 99195 sand fly specimens (59.8% males) were collected from 2011 through 2014, of which 95384 were identified at species level. Species divided by sex and month of collection in each territory are shown in S1–S9 Tables. Specimens presenting problematic identification, mainly *Phlebotomus* (*Larroussious*) females collected in Turkey and Cyprus and morphologically different from the local *L*. *infantum* vector (*P*. *tobbi* in both sites), are not included in these tables and have not been considered in further analyses. The sand fly fauna included both *Phlebotomus* and *Sergentomyia* genera; cumulatively, males were more abundant than females for most species, sites and years of collection. Furthermore, the male/female ratio was usually higher during the early periods of collection, whereas females outnumbered males in the middle of the warm season.

In sites where a combination of the two trapping methods was used, 56.1% of specimens (27295/48630) was collected by sticky traps (S10 Table). However large variations were recorded depending on sites or sand fly species: for example, yields of *P*. *perniciosus* in Spain and Portugal were almost identical by both trapping methods, whereas specimens of *P*. *perniciosus* in Italy, *P*. *neglectus* in Crete and *P*. *tobbi* in Cyprus were mostly collected by light traps. On the other hand, *Sergentomyia* specimens were almost exclusively collected by sticky traps (>90% in all sites) confirming low light attractiveness by the members of this genus.

Table 2 summarizes the distribution by country of *Phlebotomus* species identified regardless their relative abundance. From 1 to 3 species were collected in western Mediterranean sites, and from 3 to 9 species in eastern Mediterranean sites. As expected per study design, higher densities were recorded for the proven *L*. *infantum* vector species in each locality. Where two proven vector species were identified (e.g. *P*. *perniciosus* and *P*. *ariasi* in Portuguese and French sites), only the most abundant species was considered in the descriptive seasonal dynamics.

**Table 2:** *Phlebotomus* species collected in selected sites of 8 countries of the Mediterranean region. Exclusive or predominant *Leishmania infantum* vector species are marked with an asterisk.

*Phlebotomus* species	Portugal	Spain	France	Italy	Greece	Cyprus	Turkey	Georgia
Subgenus *Larroussius*								
*P*. *ariasi*	x		x*					
*P*. *perniciosus*	x*	x*	x	x*				
*P*. *neglectus*					x*			
*P*. *neglectus*/*syriacus*^a^							x	
*P*. *major* s.l.^a^							x	
*P*. *tobbi*						x*	x*	
*P*. *galilaeus*^b^						x		
*P*. *transcaucasicus*^b^							x	
*P*. *kandelakii*								x*
*P*. *wenyoni*								x
Subgenus *Paraphlebotomus*								
*P*. *sergenti*	x	x				x	x	x
*P*. *similis*					x			
*P*. *alexandri*							x	
Subgenus *Adlerius*								
*P*. *balcanicus*								x*
*P*. *halepensis*								x
*P*. *simici*							x	
Subgenus *Phlebotomus*								
*P*. *papatasi*		x			x	x	x	
Subgenus *Transphlebotomus*								
*P*. *mascittii*			x				x	

^a^ Exhibiting intermediate morphological characters within the *Phlebotomus major* group

^b^ Both species are also considered subspecies within the *Phlebotomus perfiliewi* group

### *L*. *infantum* vectors: phenology by species and site

The six *L*. *infantum* vectors marked with an asterisk in Table 2 accounted for a total of 56101 specimens collected from 2011 through 2013 (see S1–S9 Tables for details). Altogether they consisted of 8665 specimens collected in 2011 in six countries (Portugal, France, Italy, Greece, Turkey and Georgia); 28168 specimens collected in 2012 in all eight countries; and 19268 specimens collected in 2013 in six countries (Portugal, Spain, France, Greece, Cyprus and Georgia).

**Fig 2:**
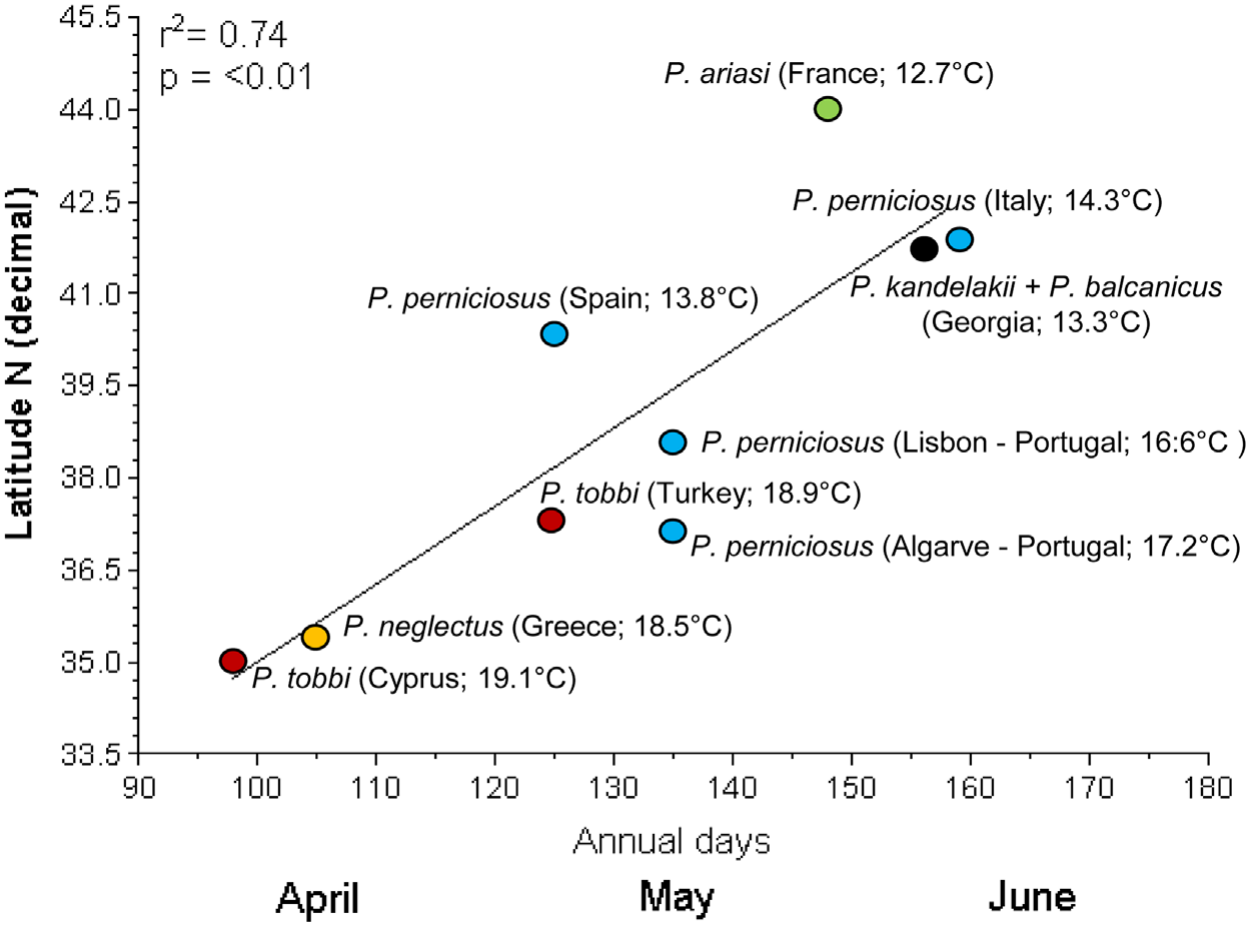
Relationship between latitude of collecting sites and period of appearance of sand flies in collections. Each species is shown by a different color, and the average annual temperature of each site is reported in parenthesis.

The main parameters for vector phenology, i.e. the period of appearance of adults in collections and the last period in which they were still collected, have been examined separately by target species and sites. Where light traps and sticky traps were used in combination, appearance or disappearance of sand flies in collections was always recorded by both methods. The relationship between the period of appearance/disappearance of vectors and latitude or average annual temperature of sites, was evaluated using data from cumulative years. When used separately in the analysis, latitude and average annual temperature gave very similar results. Fig 2 shows that a highly significant correlation exists between latitude and the first period of sand fly collection (r^2^ = 0.74; p<0.01). In the two southernmost sites of Cyprus and Crete, specimens of *P*. *tobbi* and *P*. *neglectus* were collected as early as the beginning or mid April, respectively; in four territories located at intermediate latitudes, the first specimens of *P*. *tobbi* (Turkey) and *P*. *perniciosus* (Spain and Portugal) have been captured in early or mid May, respectively; at more elevated latitudes such as those of the Italian and Georgian sites, *P*. *perniciosus*, *P*. *kandelakii* and *P*. *balcanicus* were collected starting from the first half of June. An exception to this correlation was *P*. *ariasi* from the northernmost site in France, which was collected since mid May, although only in 2011 and involving a low number of specimens (see S4 Table). When it was collected from different territories, the same vector species appeared earlier at lower latitude, and later at higher latitude: this is the case of *P*. *tobbi*, which was recorded in the Cypriot site about one month earlier than in Turkish sites, despite the two territories are located at similar altitudes. *P*. *perniciosus* was collected in different periods of May in Portugal and Spain, and in the first half of June in Italy.

**Fig 3:**
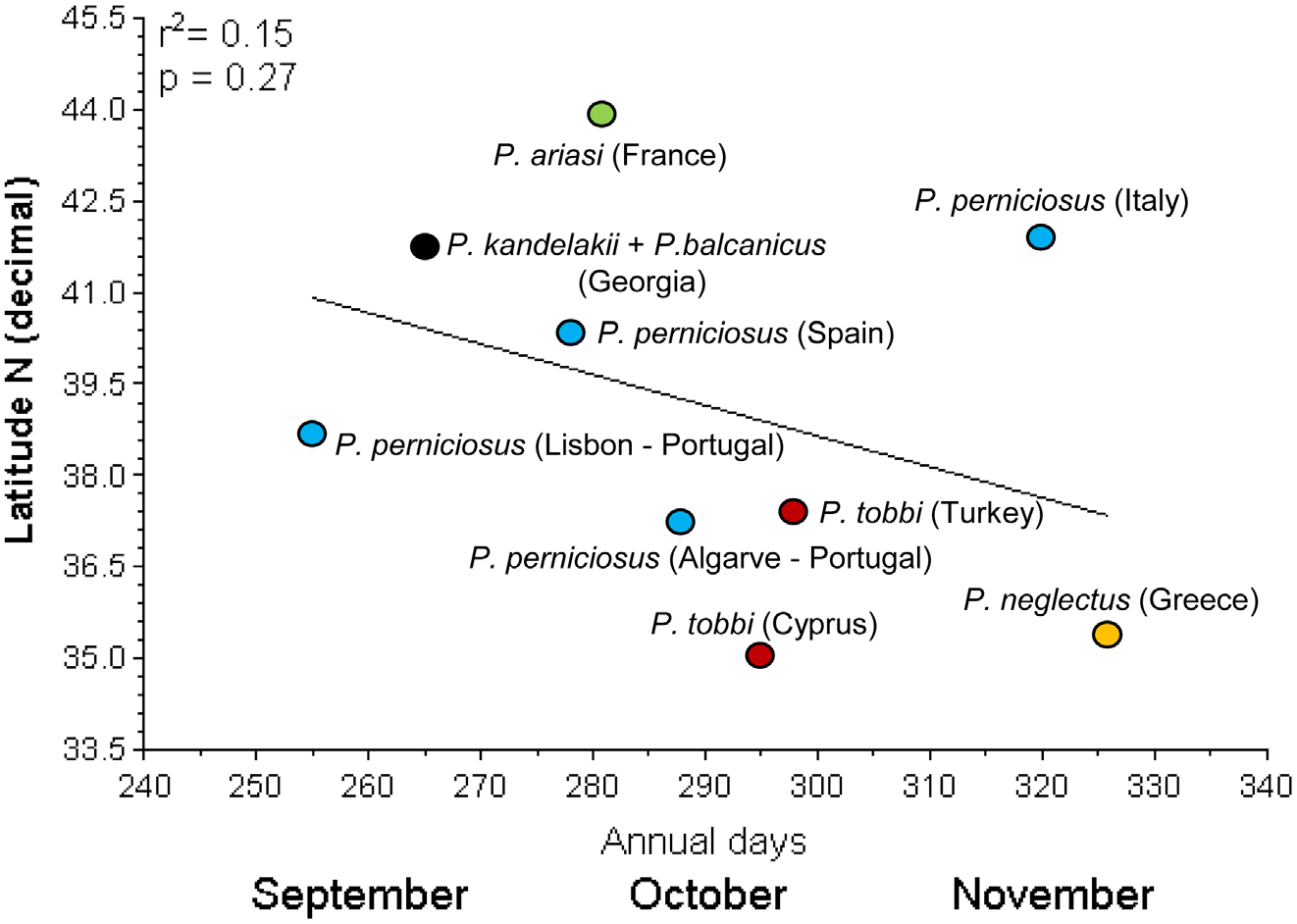
Relationship between the latitude of collecting sites and the last period of positive sand fly collection. Each species is shown by a different color.

By contrast, a similar analysis performed using the last period of sand fly collection as a variable, showed that the disappearance of adults was not significantly dictated by latitude or average annual temperature: as shown in Fig 3, each species per site appeared to be ending the activity in a period from mid September (Lisbon) to the second half of November (Crete) without significant correlation with the above parameters (r^2^ = 0.15; p = 0.27).

### Seasonal abundance by species and site

**Fig 4:**
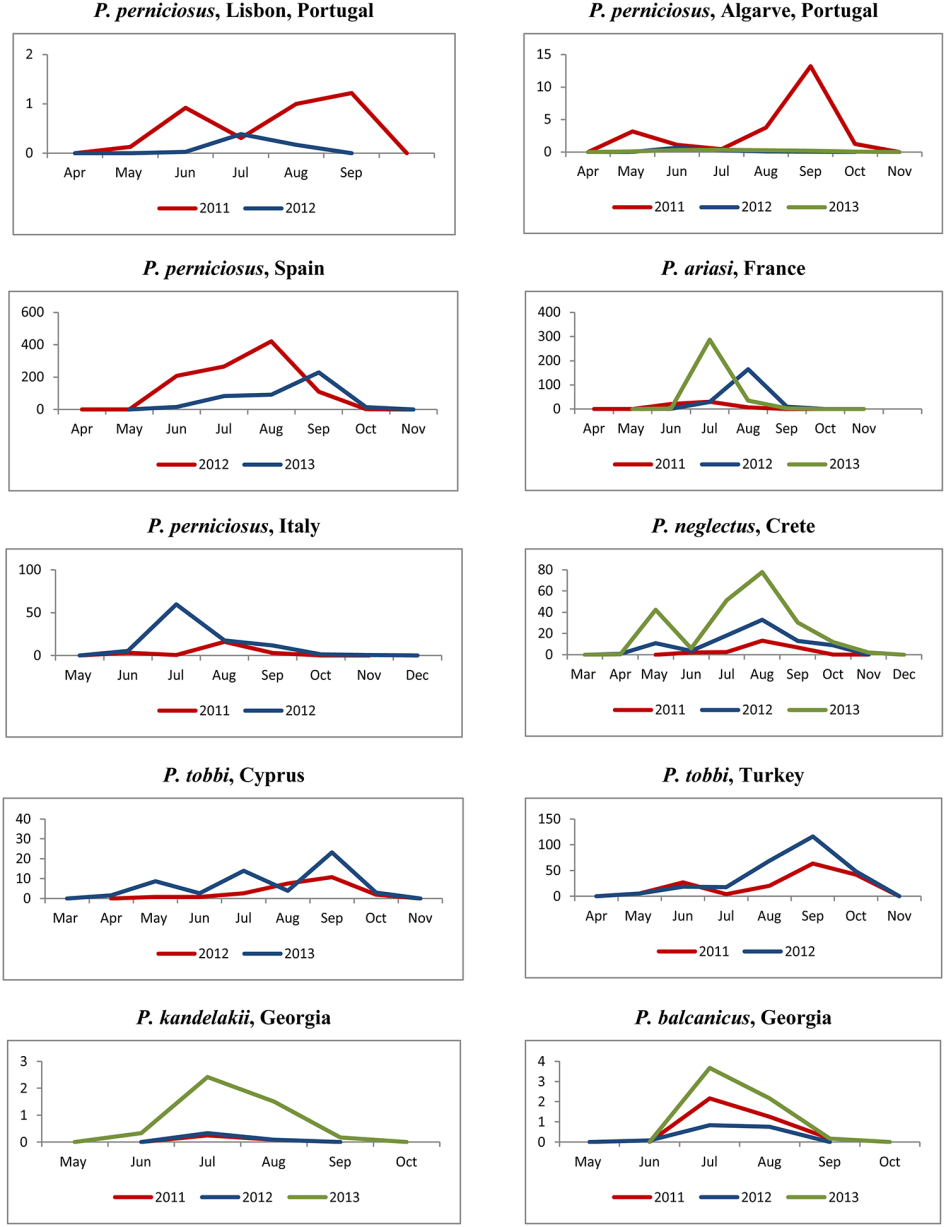
Monthly density of Mediterranean vectors of *Leishmania infantum*, 2011–2013. Density values in Y axis represent the number of specimens of the indicated vector /light trap.

The vector abundance varied between species, site and year of collection. Monthly vector densities, expressed as number of specimens/light trap, are shown for each site and year of collection in graphs of Fig 4. Total vector density in a season differed greatly between sites, from the lowest value of 0.3 *P*. *kandelakii*/ light trap/season in Tbilisi in 2011, to the highest value of ~1000 *P*. *perniciosus*/ light trap/season in Fuenlabrada in 2012. Within each site, the target species showed large annual variations in both magnitude and periodicity of density peaks which were partially influenced by local climatic events during the scheduled trapping days, such as strong wind and/or heavy rain. Peaks were rarely recorded in May (e.g. *P*. *neglectus* in Crete, 2013) or in June (e.g. *P*. *tobbi* in Turkey, 2011) whereas they were frequently observed from July through September.

**Fig 5:**
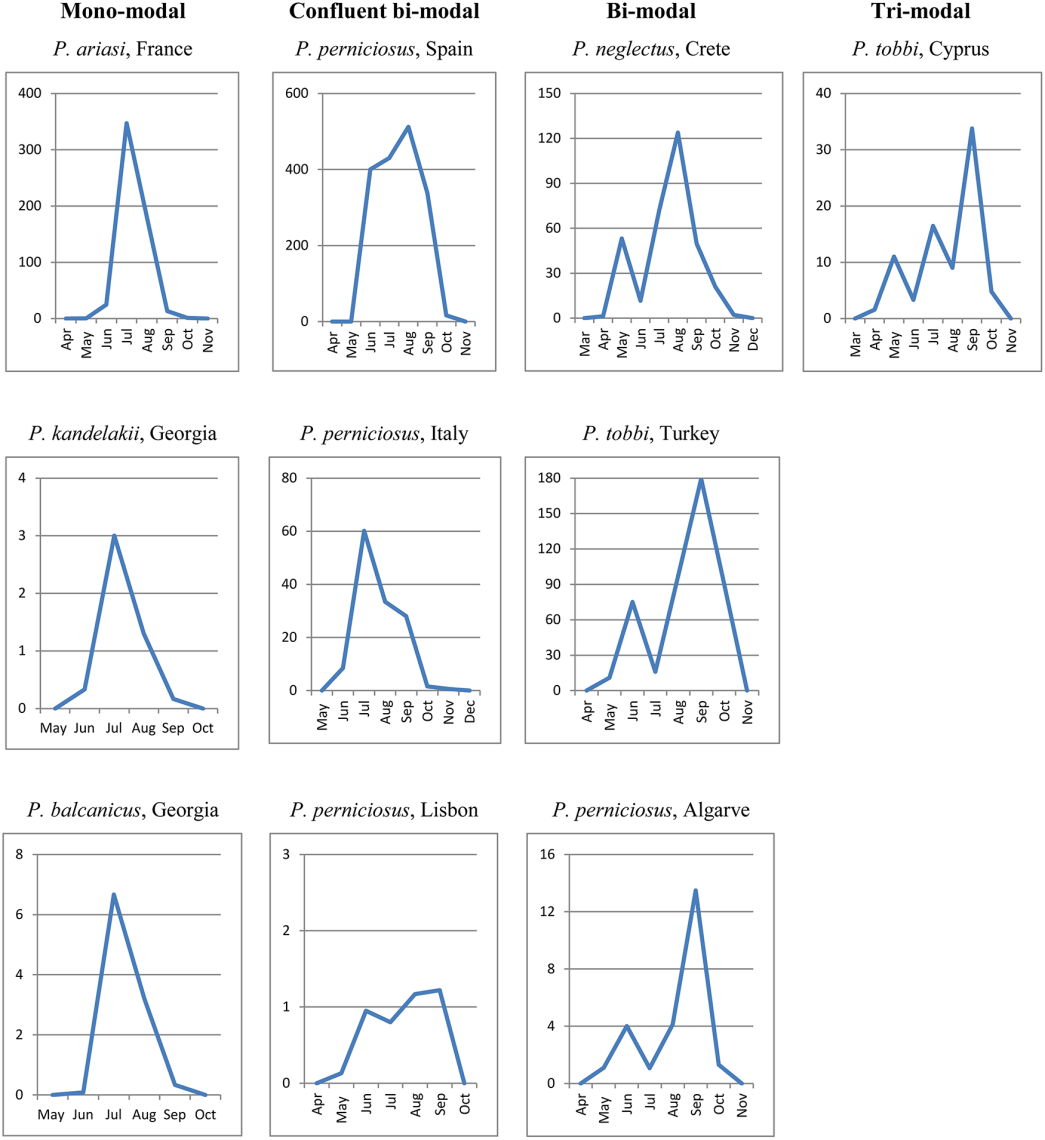
Types of abundance trends recorded in 2011–2013 for Mediterranean vectors of *Leishmania infantum*. Values in Y axis represent vector densities/light trap pooled for the years of trapping in each endemic setting

Monthly densities pooled from all years of collection, representing a more reliable picture of the potential behavior of vectors in each endemic setting, disclosed roughly four types of trends which were not found necessarily associated to a particular species (Fig 5): 1) A sharp mono-modal trend, with a single peak occurring in July-August (*P*. *ariasi*, *P*. *kandelakii* and *P*. *balcanicus*); 2) A trend much probably resulting from the confluence of 2 peaks of different or similar density encompassing a long period from June through September (*P*. *perniciosus*); 3) A sharp bi-modal trend, with a first minor peak in May-June and a major peak in August-September (*P*. *perniciosus*, *P*. *neglectus* and *P*. *tobbi*); 4) A tri-modal trend, with peaks increasing in magnitude from May through September (*P*. *tobbi*).

**Fig 6:**
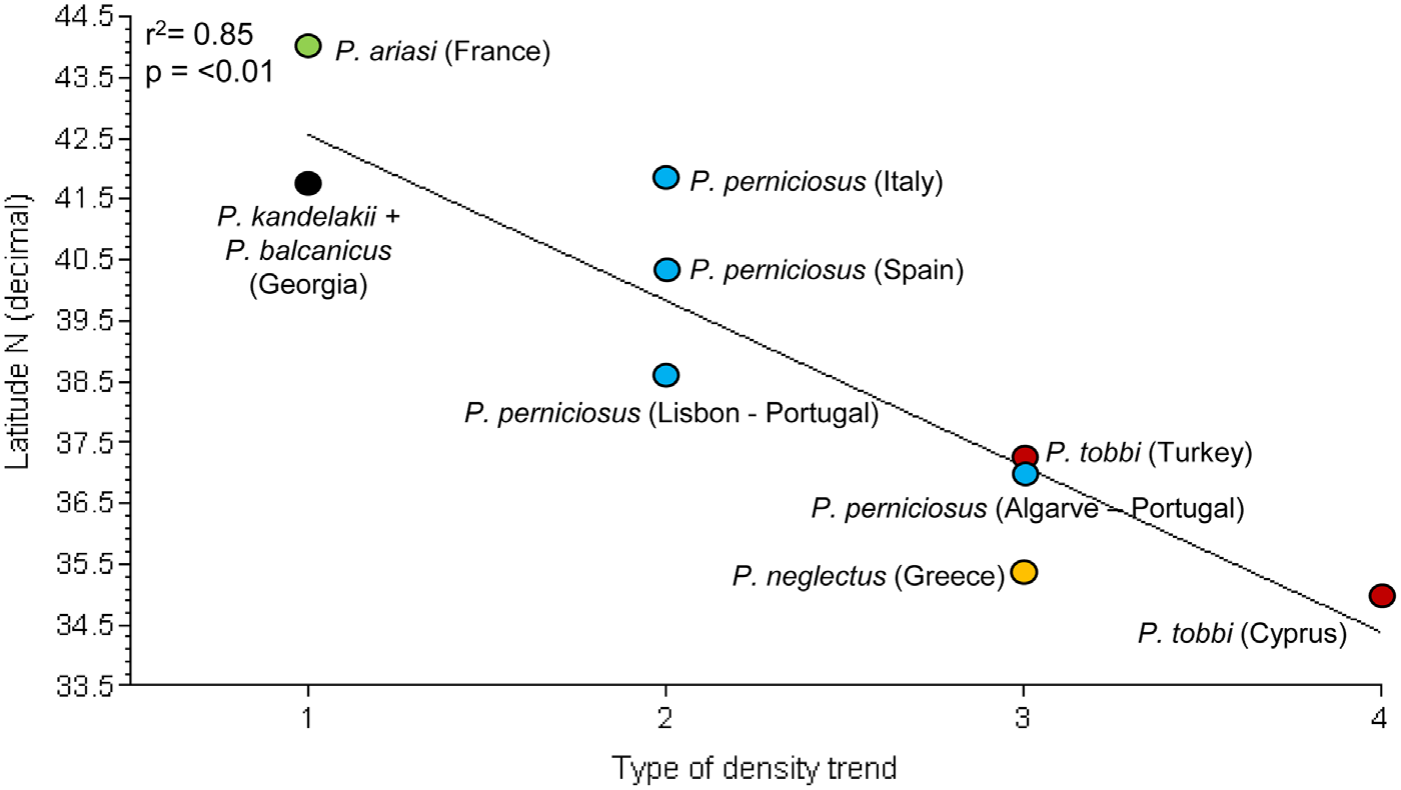
Relationship between latitude of sand fly collecting sites and type of density trend. 1: mono-modal; 2: confluence of two density peaks; 3; bi-modal; 4; tri-modal. Each species is shown by a different color.

The relationship between such types of density trend and the latitude or the average annual temperature of sites, was evaluated. When analyzed separately, again latitude and average annual temperature gave very similar results. As shown in Fig 6, a highly significant correlation was detected between latitude decrease and the increase in number of vector density peaks (r^2^ = 0.85; p <0.01). Interestingly, the trend of *P*. *tobbi* was tri-modal in the southernmost site of Cyprus, whereas it was bi-modal in Cukurova, Turkey. Analogously, the two distinct peaks observed for *P*. *perniciosus* in southern Portugal turned to be confluent at higher latitudes in the same country, as with the *P*. *perniciosus* trend in Spanish and Italian sites. The abundance patterns of French and Georgian vectors were confirmed to be typically mono-modal as repeatedly shown by historical or more recent surveys [11,56].

To verify if temperatures preceding a vector peak could influence its magnitude, temperature values recorded in relevant months were evaluated against the respective peaks of abundance; temperature/abundance interdependence was then compared between consecutive years in each site. Furthermore, the total number of vector specimens collected in a season was evaluated in respect of the average temperature recorded in the May-October period, their interdependence being also compared between years (S11 Table). We did not detect any meaningful association between temperatures registered at the beginning of the sand fly activity and the magnitude of corresponding peaks (it should be noted that no extremely high temperatures were recorded in any site which could justify estivation phenomena). For example, in the Greek site the increase of temperature from May through July was pretty similar in 2012 and 2013, however the *P*. *neglectus* peak in August 2013 was more than twice August 2012. A similar situation can be seen for *P*. *tobbi* in Cyprus in the comparison of 2012 and 2013 seasonal data. In the French site, slightly higher temperatures in May-July 2012 were associated to a *P*. *ariasi* peak much smaller than a peak occurred after lower temperatures in May-June 2013; the same is more evident for *P*. *tobbi* in Cukurova between 2011 and 2012 seasons. Finally, no relationship was detected between average summer temperatures of different years and the respective vector abundances. Sometimes lower temperatures “produced” more specimens in a season than did higher temperatures in the subsequent season (e.g. in Algarve for *P*. *perniciosus*, in France for *P*. *ariasi* or in Crete for *P*. *tobbi*); other times it was the opposite (e.g. in Italy for *P*. *perniciosus* or in Cukurova for *P*. *tobbi*).

### General overview of the seasonal dynamics of Mediterranean *L*. *infantum* vectors

**Fig 7:**
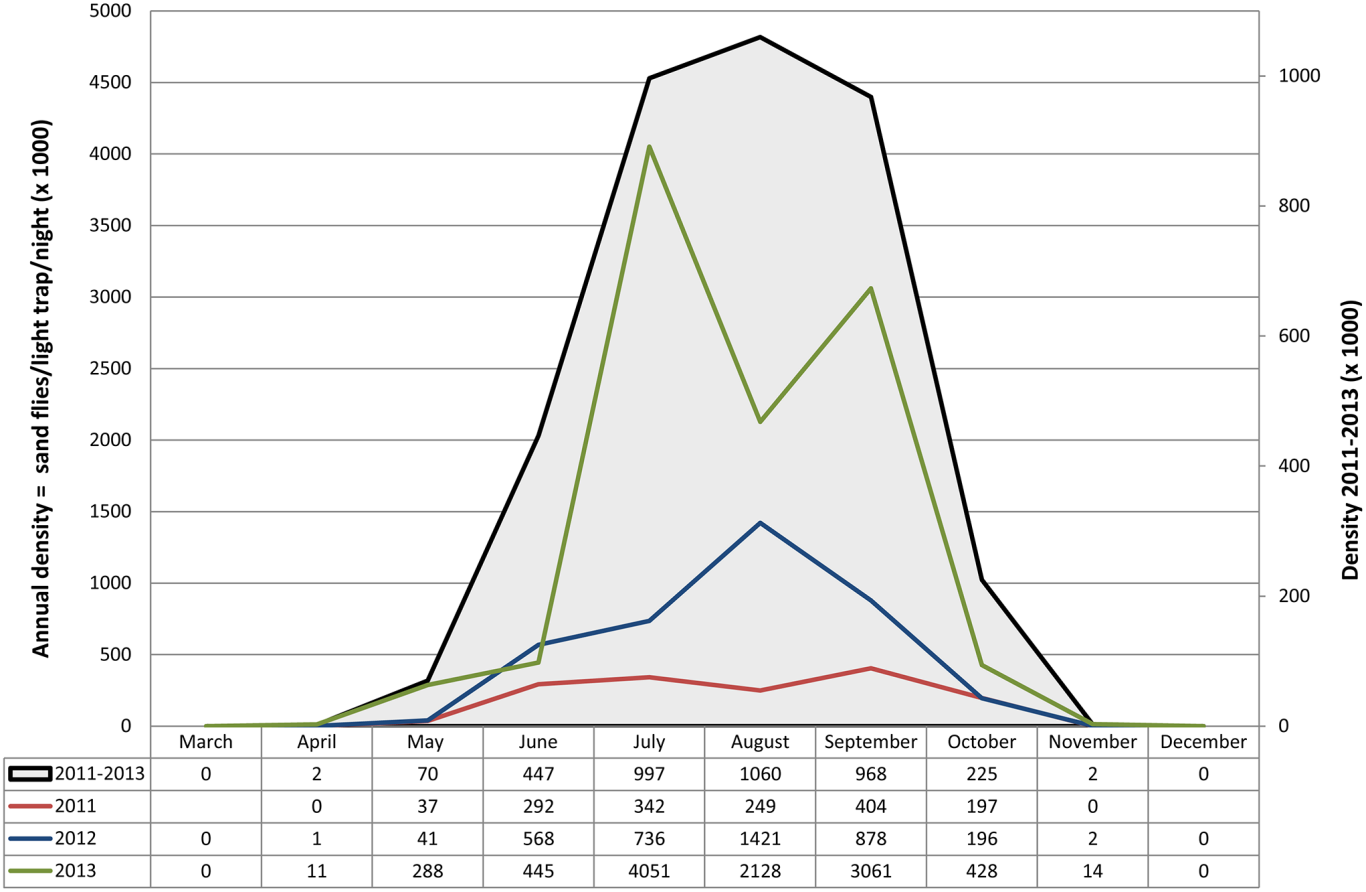
Seasonal density of *Leishmania infantum* vectors recorded in the Mediterranean region in the 2011–2013 period. Densities of 6 vector species pooled from 37 sites are shown separately for each year and for the whole period. For a better presentation of data, density values are multiplied by 1000; annual and 3-year densities are shown on different scale of values.

To answer the European health authorities about the period of potential risk of *L*.*infantum* transmission in the Mediterranean subregion, this can be inferred by the relative densities of 6 competent vector species from 37 sites over 3 years. To legitimate data pooling from different sites, total monthly catches of any vector were normalized by dividing the number of specimens by the coefficient ‘number of light traps x number of nights’ (LTxN), these parameters resulting in the overall trapping effort of 5968 LTxN in 2011, 7332 in 2012 and 1848 in 2013 (hence, 14878 LTxN for the whole 2011–2013 period). Exposure to potential infectious bites could thus be associated to a regular bell-shaped density curve having a wide peak center encompassing the July-September period, and falling between early May to late October for more than 99% of values (Fig 7). Apparently, no risk for leishmaniasis transmission took place from December through March in the years considered. This general curve results from the combination of 3 yearly patterns that look very different from each other. Probably because of generalized low yields in 2011 (a total density of 1.5 vectors/ light trap/night) no specimens were collected in April and November, nor evident density peaks were recorded during the activity period. The 2012 collections resulted in a total density of 3.8 vectors/ light trap/night, distributed in a largely confluent bi-modal trend encompassing the June-September period and having the highest peak in August. By contrast, in 2013 the total density was much higher (10.4 vectors/light trap/night) with a sharp bi-modal trend including two peaks in July and September. Despite these cumulative trends include territories with a wide range of local vector densities, there is evidence that *L*. *infantum* has been transmitted efficiently to humans and/or dogs in all of them.

### Nocturnal activity

**Fig 8:**
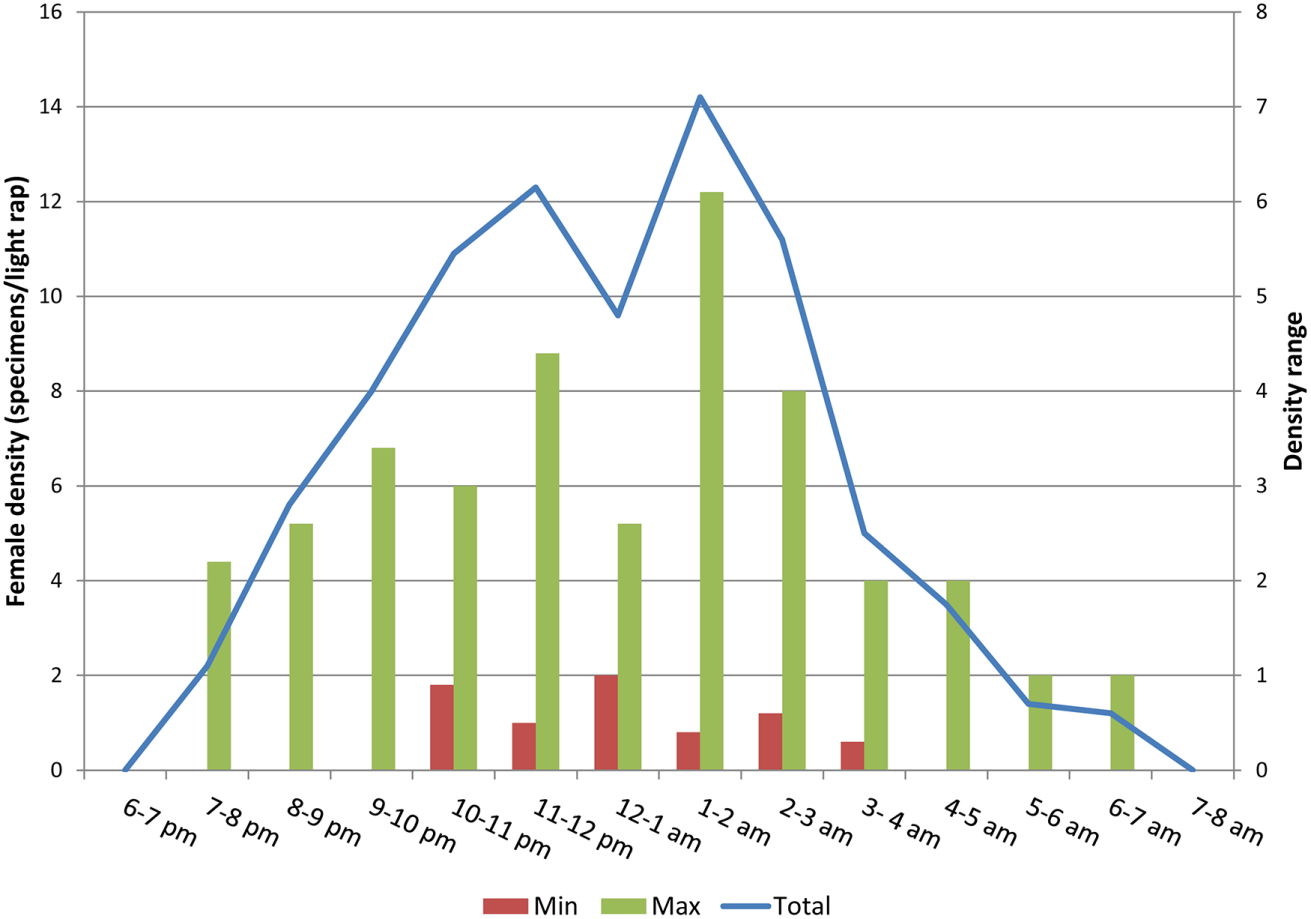
Hourly female density of *Leishmania infantum* vectors in 6 selected Mediterranean sites. Data are presented as female specimens/light trap collected per hour from all sites. Ranges represent the lowest and highest density values among all sites. Cumulative density and range are shown on different scale of values.

Hourly or bi-hourly collections were performed by light traps in one site per country, selected among those investigated for the seasonal dynamics except for a new site in the Aegean region of Turkey (Bascayir, Aydin province) and an additional site in Tbilisi, Georgia. They were typical sites for *P*. *perniciosus* (Portugal and Italy), *P*. *ariasi* (France), *P*. *tobbi* (Aegean and Cukurova regions of Turkey), *P*. *kandelakii* and *P*. *balcanicus* (Georgia). Collections were performed once a month from May or June through September or October in 2011 and 2012, respectively, in Cukurova, Turkey; once a month from June through August 2013 in Portugal; 7 days distributed among July and August 2014 in Georgia; 1 day in France in July 2011, and 1 day in Italy and Aegean Turkey in July 2012. Detailed entomological collections from each site are shown in graphs of S1 Fig, which depict a common pattern of nocturnal activity of *L*. *infantum* vectors. Furthermore, hourly activity were found similar in multiple collections performed in different months in the same site. To complement the above information on the temporal risk of *L*.*infantum* transmission in the Mediterranean subregion, a general picture of the nocturnal behavior of females was produced, based on data from 1167 sand flies (40.5% females) collected through a trapping effort of 36 light traps/night. The mean female density/light trap in a night was 13, with a range among sites of 6–22. The hourly density of vectors is shown in Fig 8, which also reports on the density range among sites. Depending on the local vector abundance, females activity started in a range between 7 and 10 pm, and ended in a range between 4 and 7 am, with slight variations among species and sites (for example, the few *P*. *balcanicus* females collected in Tibilisi ended the activity at 2 am). Peaks of activity (12–14 female density) were found between 11 pm and 2 am, whereas the activity decreased sharply after 3 am.

## Discussion

A large retrospective meta-analysis of phenology data sets collected on animals and plants over the past few decades has indicated an advance of spring/summer in Europe [24]. In particular, invertebrates have shown an earlier shift in phenological indicators of about 4 days over 30 years of observations, at latitudes of northern hemisphere that include the Mediterranean basin [57]. Because the global average temperature is projected to rise at a rapid rate [32], it is expected that earlier shifts will increase in magnitude in the near-mid future. As regards Mediterranean phlebotomine sand flies, a retrospective analysis of phenology data sets published over the past 50 years did not result in meaningful conclusions due to the limitations highlighted in the introduction of this paper. Of our investigated sites, much of previous information was left unpublished or the data were relatively recent, such as those from Georgia [11] or eastern Turkey sites [58]. As expected, in these sites the main parameters of seasonal dynamics were found unchanged. In the French location of Roquedur-le-haut, no phenological variations have occurred since the collections performed in 1977 by Rioux et al. [59] as compared to our 2011 trapping: in both years, the emergence of adults was recorded on May. Hence, our data from several georeferenced sites may represent a robust starting point for continuing surveillance on sand fly dynamics that may impact on leishmaniasis transmission in the near future. Among basic entomological parameters, the unequivocal identification of species and their ascertained role as leishmaniasis vectors is important. Therefore species so far unproven to transmit *L*. *infantum* were excluded from our analyses.

Our findings confirm historical observations that temperature—whose magnitude is negatively correlated with latitude—is a major determinant for the activity start of leishmaniasis vectors. It is well established that during cold months sand flies undergo diapause as fourth larval stage (L4). In laboratory-reared species, at low temperature conditions a diapause period may run even 9 months, and only after the temperature is raised to a certain level L4 larvae pupate and adults emerge within two or three weeks [60]. Thus, in our Mediterranean sites the periods of pupation and adults emergence were much probably dependent on generalized temperature increase to which L4 larvae were exposed in their natural environment, approximately in March, April or May in sites where adults were firstly collected in April, May or June, respectively. The emergence of some vectors (*P*. *tobbi* and *P*. *neglectus*) as early as April in southern Mediterranean latitudes (around 35° N) is not surprising, as *P*. *perniciosus* was found the behave in the same way at similar latitudes (around 37° N, e.g. in Tunis [61], Almeria [28] and Catania [31]).

On the other hand, we have shown that the end of adults activity may occur at different months, from September through November, independently from latitude, average annual temperature, or temperatures recorded in the sites during the activity season. Several investigations on Mediterranean *L*. *infantum* vectors reported on such time interval for the last sand fly-positive collections; very recently, specimens of *P*. *perniciosus* were collected in Catania even in early December [31]. A careful analysis of literature with particular regard to the latitude of collecting sites suggests that our findings are well supported, as no apparent association could be detected between this parameter and the disappearance of adults in collections. It suggests that for this stage of life cycle the dependence on average annual temperature is less pronounced. Laboratory observations have shown that adults are not much sensitive to temperature changes as they are larval stages: lower temperatures slow down sand fly metabolism and increase survival time especially of blood-fed females; periods of developmental phases become longer as regards defecation, oviposition and eggs hatch [62]. These processes may be variable from one site to the other, and may depend on the initial larval/adult population size, local environmental variables and on less investigated parameters interfaced with temperature such as endogenous clocks sensitive to photoperiod [63,64].

Vector densities varied greatly between different sites, and in most of them seasonal and monthly density patterns were also very different between years. A recent analysis of historical *P*. *ariasi* data has confirmed that large differences in abundance can be found between close collecting stations within the same territory, without appreciable relationship with major phytoecological variables [56]. Local weather events limited in time, such as strong wind and/or heavy rain, were found to affect the productivity of adults in our scheduled periods of collection; this fact could have offered a biased picture of the potential phlebotomine behaviour in case the investigations were performed during a single season only. In the search for causes underlying variations in annual density patterns, we could not detect any association between temperatures recorded during the collection period and the concomitant occurrence/magnitude of density peaks or the total vector abundance. This would suggest, rather, that winter (or, in general, the whole annual mean temperature) may affect the relative density of adults emerging in the subsequent spring/summer, most probably having an impact on the survival rate of overwintering larval populations. Actually, the occurrence of multiple density peaks was significantly correlated with sites located at lower latitudes and having higher annual temperatures. The earlier peaks, representing the first emerging adult generation, have always been smaller than the subsequent peak(s), which may indicate a sequence of summer generations with increasing abundance. The occurrence of mono-modal peaks was repeatedly confirmed in French *P*. *ariasi* and in Georgian vectors [11, 56]. In contrast, different patterns of density have been reported for *P*. *perniciosus* from different sites, or from the same site in different years. A sharp bi-modal trend of this species, peaking in July and September, was consistently found over the years in Mediterranean sites located at low latitudes, such as Tunis [61], Almeria [28], Algarve area [65] and Catania [31]. In intermediate or higher latitudes, instead, single large peaks or two partially confluent peaks of *P*. *perniciosus* encompassing the June/July-September period, have been recorded (e.g. Torres Novas, central Portugal [66]; Régua, north Portugal [67]; Madrid province, central Spain [68]; and Putignano, Italy [69]). It should be noted, however, that at these latitudes there are examples of variable *P*. *pernicosus* density patterns recorded in different years from the same sites. In two consecutive years, a wide mono-modal trend and two partially overlapping peaks, respectively, were reported in Rome province [29]; analogously, a large mono-modal and a sharp bi-modal trend where shown in Marseille area after 2 years, respectively [70]. Hence, in sites located at intermediate and higher latitudes the potential behavior of *P*. *perniciosus* can be better depicted from multi-seasonal data as having a wide confluent pattern of density, which can be subject to annual variations within its range. Finally, for the first time a tri-modal peak was observed in a *L*. *infantum* vector (*P*. *tobbi*, Cyprus). After emergence of this species in April, density peaks have been recorded in May, July and September, which is compatible with a series of 2 summer generations.

The nocturnal activity of phlebotomine vectors is generated by the circadian clock that coordinates feeding activities important for the dynamics of *Leishmania* transmission [63]. While we do not expect that the circadian rhythm will be much influenced by the rise in global average temperature, we consider our findings as a useful complement to evaluate and predict leishmaniasis risk associated with sand fly activity and abundance. We recorded a common pattern of vector females activity that, at least in places with higher vector abundance, increased shortly after sunset and continued until just after sunrise. However the main period of activity was found between 11 pm and 2 am for all vector species, which is an important information for the evaluation of exposure risk to *L*. *infantum*. The female population was collected by light traps, so that it included specimens with different activity behaviors such as sugar feeding, host seeking, blood feeding, mating, and oviposition. We did not determine the parous rate of females, which could be important to evaluate the behavior of potentially *Leishmania*-infected flies. Recently, it was shown that parous and nulliparous females of *P*. *orientalis* (a member of the *Larroussius* subgenus) exhibit different periodicity of nocturnal abundance [71].

In conclusions, we found that cumulative collections performed over consecutive years can provide homogeneous patterns of the potential behavior of leishmaniasis vectors in selected sites, which we propose may represent sentinel areas for future monitoring.

Considering both, the relative adults densities and the low probability of females harboring parasite infections in earliest periods of activity, the highest potential risk for *L*. *infantum* transmission in the Mediterranean subregion may be identified during the months from June through October (97% relative vector density) in the investigated period. We can also conclude that such risk was not equally distributed throughout the region, since earlier emergence and density waves of sand flies were more frequent in southern territories.

## Supporting Information

S1 Fig Hourly or bi-hourly CDC-light trap collection of *L*. *infantum* vector specimens in 6 selected Mediterranean sites. (TIF)

S1 Table Phlebotomine sand fly species collected in 11 sites of the Lisbon Metropolitan region, Portugal. (DOCX)

S2 Table Phlebotomine sand fly species collected in 11 sites of the Algarve region, Portugal. (DOCX)

S3 Table Phlebotomine sand fly species collected in Fuenlabrada, Spain. (DOCX)

S4 Table Phlebotomine sand fly species collected in Roquedur-le-haut, France. (DOCX)

S5 Table Phlebotomine sand fly species collected in Frascati, Italy. (DOCX)

S6 Table Phlebotomine sand fly species collected in Fodele, Crete, Greece. (DOCX)

S7 Table Phlebotomine sand fly species collected in Steni, Cyprus. (DOCX)

S8 Table Phlebotomine sand fly species collected in 6 sites of Cukurova region, Turkey. (DOCX)

S9 Table Phlebotomine sand fly species collected in 2 sites of Tbilisi, Georgia. (DOCX)

S10 Table Results of phlebotomine sand fly collections in sites where miniature CDC light trapping (CDC) was regularly associated with sticky paper trapping (ST). (DOCX)

S11 Table Comparison of temperatures and vector abundances recorded in each site between different years. Examples are given of the interdependency between i) average monthly temperatures registered at the beginning of the sand fly activity and magnitude of peaks; and ii) average summer temperatures and total vector abundance. (PDF)
