# Supplementary figures and images for: Seasonal Dynamics of Phlebotomine Sand Fly Species Proven Vectors of Mediterranean Leishmaniasis Caused by Leishmania infantum
Source: PLoS Negl Trop Dis. 2016 Feb 22;10(2):e0004458. doi: 10.1371/journal.pntd.0004458 (PMC4762948; doi:10.1371/journal.pntd.0004458)

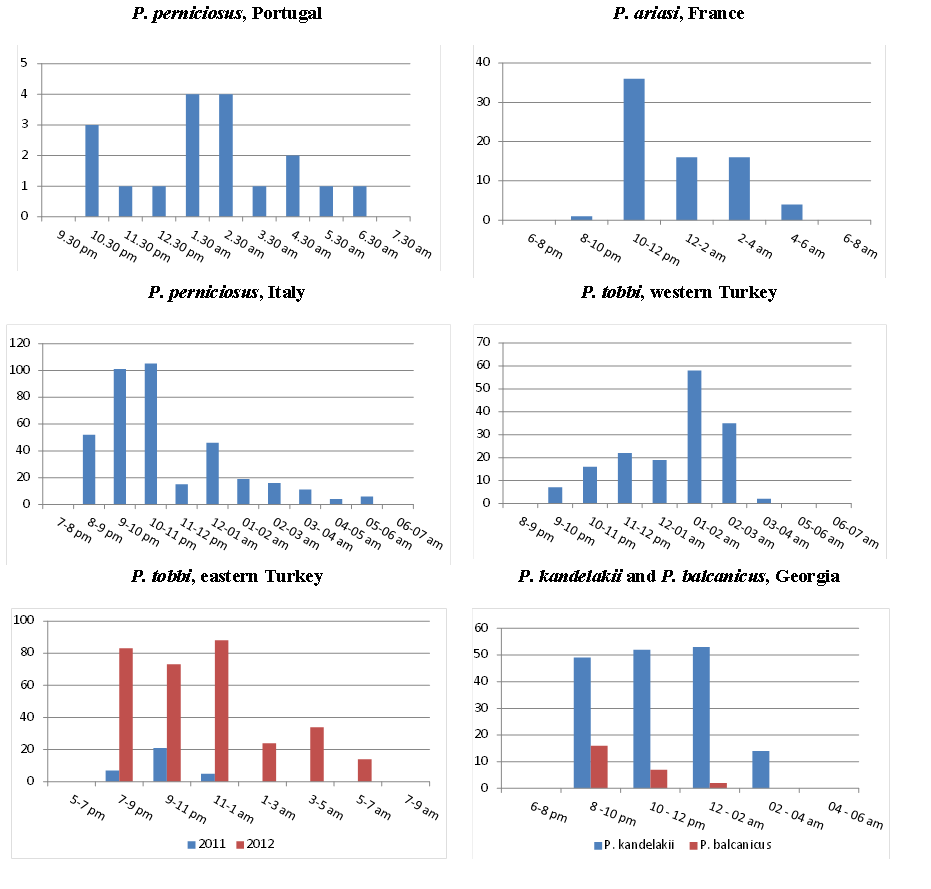

Supplement: S1 Fig — (TIF) [file pntd.0004458.s001.tif]
