# Supplementary material for: Seasonal Dynamics of Phlebotomine Sand Fly Species Proven Vectors of Mediterranean Leishmaniasis Caused by Leishmania infantum
Source: PLoS Negl Trop Dis. 2016 Feb 22;10(2):e0004458. doi: 10.1371/journal.pntd.0004458 (PMC4762948; doi:10.1371/journal.pntd.0004458)
Supplement: S1 Table — (DOCX) [file pntd.0004458.s002.docx]

Table S1. Phlebotomine sand fly species collected in 11 sites of the Lisbon Metropolitan region, Portugal

| Year | Month | *P. ariasi* | | Total | *S. minuta* | | Total | *P. perniciosus* | | Total | *P. sergenti* | | Total |
| --- | --- | --- | --- | --- | --- | --- | --- | --- | --- | --- | --- | --- | --- |
|  |  | Female | Male |  | Female | Male |  | Female | Male |  | Female | Male |  |
| 2011 | April | 0 | 0 | 0 | 0 | 0 | 0 | 0 | 0 | 0 | 0 | 0 | 0 |
|  | May | 10 | 15 | 25 | 0 | 0 | 0 | 1 | 5 | 6 | 0 | 0 | 0 |
|  | June | 6 | 9 | 15 | 1 | 2 | 3 | 6 | 31 | 37 | 0 | 2 | 2 |
|  | July | 6 | 24 | 31 | 7 | 14 | 21 | 5 | 10 | 15 | 3 | 5 | 8 |
|  | August | 19 | 81 | 100 | 4 | 18 | 22 | 3 | 34 | 37 | 0 | 1 | 1 |
|  | September | 15 | 44 | 59 | 5 | 4 | 9 | 11 | 37 | 48 | 0 | 0 | 0 |
|  | October | 1 | 20 | 21 | 0 | 0 | 0 | 0 | 0 | 0 | 0 | 0 | 0 |
|  | November | 0 | 0 | 0 | 0 | 0 | 0 | 0 | 0 | 0 | 0 | 0 | 0 |
|  | Total | 57 | 193 | 250 | 17 | 38 | 55 | 26 | 117 | 143 | 3 | 8 | 11 |
| 2012 | April | 0 | 0 | 0 | 0 | 0 | 0 | 0 | 0 | 0 | 0 | 0 | 0 |
|  | May | 0 | 0 | 0 | 0 | 1 | 1 | 0 | 0 | 0 | 0 | 0 | 0 |
|  | June | 0 | 2 | 2 | 1 | 10 | 11 | 0 | 1 | 1 | 0 | 0 | 0 |
|  | July | 0 | 0 | 0 | 1 | 6 | 7 | 12 | 10 | 22 | 3 | 1 | 4 |
|  | August | 2 | 0 | 2 | 4 | 2 | 6 | 8 | 0 | 8 | 0 | 0 | 0 |
|  | September | 1 | 0 | 1 | 0 | 0 | 0 | 0 | 0 | 0 | 0 | 0 | 0 |
|  | October | 0 | 0 | 0 | 0 | 0 | 0 | 0 | 0 | 0 | 0 | 0 | 0 |
|  | November | 0 | 0 | 0 | 0 | 0 | 0 | 0 | 0 | 0 | 0 | 0 | 0 |
|  | Total | 3 | 2 | 5 | 6 | 19 | 25 | 20 | 11 | 31 | 1 | 3 | 4 |
