# Supplementary material for: Seasonal Dynamics of Phlebotomine Sand Fly Species Proven Vectors of Mediterranean Leishmaniasis Caused by Leishmania infantum
Source: PLoS Negl Trop Dis. 2016 Feb 22;10(2):e0004458. doi: 10.1371/journal.pntd.0004458 (PMC4762948; doi:10.1371/journal.pntd.0004458)
Supplement: S2 Table — (DOCX) [file pntd.0004458.s003.docx]

Table S2. Phlebotomine sand fly species collected in 11 sites of the Algarve region, Portugal

| Year | Month | *P. ariasi* | | Total | *S. minuta* | *P. perniciosus* | | Total | *P. sergenti* |
| --- | --- | --- | --- | --- | --- | --- | --- | --- | --- |
|  |  | Female | Male |  |  | Female | Male |  |  |
| 2011 | April | 0 | 0 | 0 | 0 | 0 | 0 | 0 | 0 |
|  | May | 17 | 4 | 21 | 32 | 15 | 27 | 42 | 1 |
|  | June | 0 | 0 | 0 | 80 | 31 | 28 | 59 | 21 |
|  | July | 0 | 0 | 0 | 438 | 8 | 21 | 29 | 26 |
|  | August | 3 | 1 | 4 | 689 | 86 | 76 | 162 | 18 |
|  | September | 8 | 0 | 8 | 507 | 218 | 269 | 487 | 13 |
|  | October | 0 | 0 | 0 | 52 | 26 | 39 | 65 | 0 |
|  | November | 0 | 0 | 0 | 0 | 0 | 0 | 0 | 0 |
|  | Total | 28 | 5 | 33 | 1798 | 384 | 460 | 844 | 79 |
| 2012 | April | 0 | 0 | 0 | 0 | 0 | 0 | 0 | 0 |
|  | May | 0 | 0 | 0 | 130 | 2 | 33 | 35 | 0 |
|  | June | 1 | 0 | 1 | 117 | 24 | 112 | 136 | 2 |
|  | July | 0 | 0 | 0 | 362 | 11 | 81 | 92 | 7 |
|  | August | 0 | 1 | 1 | 179 | 5 | 69 | 74 | 4 |
|  | September | 3 | 0 | 3 | 110 | 9 | 87 | 96 | 3 |
|  | October | 0 | 0 | 0 | 19 | 3 | 0 | 3 | 0 |
|  | November | 0 | 0 | 0 | 0 | 0 | 0 | 0 | 0 |
|  | Total | 4 | 1 | 5 | 917 | 54 | 382 | 436 | 16 |
| 2013 | April | 0 | 0 | 0 | 0 | 0 | 0 | 0 | 0 |
|  | May | 2 | 1 | 3 | 32 | 4 | 30 | 34 | 0 |
|  | June | 0 | 1 | 1 | 47 | 2 | 50 | 52 | 10 |
|  | July | 0 | 1 | 1 | 107 | 11 | 38 | 49 | 13 |
|  | August | 0 | 0 | 0 | 124 | 8 | 33 | 41 | 5 |
|  | September | 0 | 1 | 1 | 201 | 4 | 119 | 123 | 1 |
|  | October | 0 | 0 | 0 | 21 | 3 | 52 | 55 | 2 |
|  | November | 0 | 0 | 0 | 0 | 0 | 0 | 0 | 0 |
|  | Total | 2 | 4 | 6 | 532 | 32 | 322 | 354 | 31 |
