# Supplementary material for: Seasonal Dynamics of Phlebotomine Sand Fly Species Proven Vectors of Mediterranean Leishmaniasis Caused by Leishmania infantum
Source: PLoS Negl Trop Dis. 2016 Feb 22;10(2):e0004458. doi: 10.1371/journal.pntd.0004458 (PMC4762948; doi:10.1371/journal.pntd.0004458)
Supplement: S3 Table — (DOCX) [file pntd.0004458.s004.docx]

Table S3. Phlebotomine sand fly species collected in Fuenlabrada, Spain

| Year | Month | *S. minuta* (*) | | Total  (**) | *P. perniciosus* (*) | | Total  (**) | *P. sergenti* (*) | | Total  (**) | *P. papatasi* (*) | | Total  (**) |
| --- | --- | --- | --- | --- | --- | --- | --- | --- | --- | --- | --- | --- | --- |
|  |  | Female | Male |  | Female | Male |  | Female | Male |  | Female | Male |  |
| 2012 | April | 0 | 0 | 0 | 0 | 0 | 0 | 0 | 0 | 0 | 0 | 0 | 0 |
|  | May | 0 | 0 | 1 | 0 | 2 | 4 | 0 | 0 | 0 | 0 | 0 | 0 |
|  | June | 3 | 16 | 166 | 1088 | 573 | 3289 | 0 | 0 | 0 | 0 | 0 | 0 |
|  | July | 34 | 110 | 1155 | 1527 | 600 | 3330 | 1 | 0 | 1 | 0 | 0 | 0 |
|  | August | 73 | 134 | 1328 | 1071 | 2296 | 5007 | 1 | 0 | 1 | 0 | 0 | 0 |
|  | September | 32 | 44 | 549 | 603 | 272 | 2391 | 0 | 0 | 0 | 0 | 0 | 0 |
|  | October | 0 | 0 | 20 | 4 | 10 | 80 | 0 | 0 | 0 | 0 | 0 | 0 |
|  | November | 0 | 0 | 0 | 0 | 0 | 0 | 0 | 0 | 0 | 0 | 0 | 0 |
|  | Total | 142 | 304 | 3219 | 4293 | 3753 | 14101 | 2 | 0 | 2 | 0 | 0 | 0 |
| 2013 | April | 0 | 0 | 0 | 0 | 0 | 0 | 0 | 0 | 0 | 0 | 0 | 0 |
|  | May | 0 | 0 | 0 | 0 | 0 | 0 | 0 | 0 | 0 | 0 | 0 | 0 |
|  | June | 12 | 12 | 107 | 49 | 77 | 367 | 0 | 0 | 0 | 0 | 0 | 0 |
|  | July | 29 | 20 | 754 | 303 | 362 | 1582 | 0 | 0 | 0 | 0 | 1 | 1 |
|  | August | 70 | 77 | 1551 | 227 | 503 | 1596 | 0 | 0 | 0 | 0 | 0 | 0 |
|  | September | 41 | 43 | 922 | 753 | 1077 | 4929 | 0 | 0 | 0 | 0 | 0 | 0 |
|  | October | 1 | 3 | 50 | 52 | 66 | 548 | 0 | 0 | 0 | 0 | 0 | 0 |
|  | November | 0 | 0 | 0 | 0 | 0 | 0 | 0 | 0 | 0 | 0 | 0 | 0 |
|  | Total | 153 | 155 | 3384 | 1384 | 2085 | 9022 | 0 | 0 | 0 | 0 | 1 | 1 |

(*) CDC light traps only

(**) CDC light traps + sticky traps
