# Supplementary material for: Seasonal Dynamics of Phlebotomine Sand Fly Species Proven Vectors of Mediterranean Leishmaniasis Caused by Leishmania infantum
Source: PLoS Negl Trop Dis. 2016 Feb 22;10(2):e0004458. doi: 10.1371/journal.pntd.0004458 (PMC4762948; doi:10.1371/journal.pntd.0004458)
Supplement: S4 Table — (DOCX) [file pntd.0004458.s005.docx]

Table S4. Phlebotomine sand fly species collected in Roquedur-le-haut, France

| Year | Month | *P. ariasi* | | Total | *S. minuta* | | Total | *P. perniciosus* | | Total | *P. mascittii* | | Total |
| --- | --- | --- | --- | --- | --- | --- | --- | --- | --- | --- | --- | --- | --- |
|  |  | Female | Male |  | Female | Male |  | Female | Male |  | Female | Male |  |
| 2011 | April | 0 | 0 | 0 | 0 | 0 | 0 | 0 | 0 | 0 | 0 | 0 | 0 |
|  | May | 3 | 28 | 31 | 2 | 3 | 5 | 0 | 0 | 0 | 0 | 0 | 0 |
|  | June | 216 | 478 | 694 | 21 | 21 | 42 | 1 | 4 | 5 | 0 | 0 | 0 |
|  | July | 666 | 1054 | 1720 | 72 | 89 | 161 | 8 | 25 | 33 | 1 | 0 | 1 |
|  | August | 44 | 321 | 365 | 2 | 15 | 17 | 0 | 0 | 0 | 2 | 0 | 2 |
|  | September | 5 | 51 | 56 | 12 | 21 | 33 | 1 | 0 | 1 | 0 | 0 | 0 |
|  | October | 0 | 0 | 0 | 0 | 0 | 0 | 0 | 0 | 0 | 0 | 0 | 0 |
|  | November | 0 | 0 | 0 | 0 | 0 | 0 | 0 | 0 | 0 | 0 | 0 | 0 |
|  | Total | 934 | 1932 | 2866 | 109 | 149 | 258 | 10 | 29 | 39 | 3 | 0 | 3 |
| 2012 | May | 0 | 0 | 0 | 0 | 0 | 0 | 0 | 0 | 0 | 0 | 0 | 0 |
|  | June | 11 | 124 | 135 | 19 | 78 | 97 | 0 | 2 | 2 | 0 | 0 | 0 |
|  | July | 392 | 322 | 714 | 1 | 5 | 6 | 0 | 4 | 4 | 0 | 0 | 0 |
|  | August | 2322 | 684 | 3006 | 76 | 67 | 143 | 0 | 13 | 13 | 2 | 0 | 2 |
|  | September | 93 | 523 | 616 | 20 | 54 | 74 | 0 | 2 | 2 | 5 | 0 | 5 |
|  | October | 10 | 21 | 31 | 0 | 0 | 0 | 0 | 0 | 0 | 0 | 0 | 0 |
|  | November | 2 | 4 | 6 | 0 | 0 | 0 | 0 | 0 | 0 | 0 | 0 | 0 |
|  | December | 0 | 0 | 0 | 0 | 0 | 0 | 0 | 0 | 0 | 0 | 0 | 0 |
|  | Total | 2830 | 1678 | 4508 | 116 | 204 | 320 | 0 | 21 | 21 | 7 | 0 | 7 |
| 2013 | May | 0 | 0 | 0 | 0 | 0 | 0 | 0 | 0 | 0 | 0 | 0 | 0 |
|  | June | 35 | 282 | 317 | 27 | 60 | 87 | 0 | 2 | 2 | 0 | 0 | 0 |
|  | July | 2988 | 2180 | 5168 | 67 | 62 | 129 | 0 | 8 | 8 | 2 | 1 | 3 |
|  | August | 324 | 1155 | 1479 | 68 | 93 | 161 | 1 | 4 | 5 | 4 | 0 | 4 |
|  | September | 14 | 85 | 99 | 0 | 2 | 2 | 0 | 0 | 0 | 0 | 0 | 0 |
|  | October | 0 | 2 | 2 | 0 | 0 | 0 | 0 | 0 | 0 | 0 | 0 | 0 |
|  | November | 0 | 0 | 0 | 0 | 0 | 0 | 0 | 0 | 0 | 0 | 0 | 0 |
|  | Total | 3361 | 3704 | 7065 | 162 | 217 | 379 | 1 | 14 | 15 | 6 | 1 | 7 |
