# Supplementary material for: Seasonal Dynamics of Phlebotomine Sand Fly Species Proven Vectors of Mediterranean Leishmaniasis Caused by Leishmania infantum
Source: PLoS Negl Trop Dis. 2016 Feb 22;10(2):e0004458. doi: 10.1371/journal.pntd.0004458 (PMC4762948; doi:10.1371/journal.pntd.0004458)
Supplement: S5 Table — (DOCX) [file pntd.0004458.s006.docx]

Table S5. Phlebotomine sand fly species collected in Frascati, Italy

| Year | Month | *S. minuta* | | Total | *P. perniciosus* | | Total |
| --- | --- | --- | --- | --- | --- | --- | --- |
|  |  | Female | Male |  | Female | Male |  |
| 2011 | April | 0 | 0 | 0 | 0 | 0 | 0 |
|  | May | 3 | 5 | 8 | 0 | 0 | 0 |
|  | June | 93 | 155 | 248 | 57 | 113 | 170 |
|  | July | 382 | 204 | 586 | 8 | 21 | 29 |
|  | August | 424 | 798 | 1222 | 56 | 137 | 193 |
|  | September | 137 | 254 | 391 | 17 | 32 | 49 |
|  | October | 12 | 11 | 23 | 3 | 1 | 4 |
|  | November | 0 | 0 | 0 | 0 | 0 | 0 |
|  | Total | 1051 | 1427 | 2478 | 141 | 304 | 445 |
| 2012 | April | 0 | 0 | 0 | 0 | 0 | 0 |
|  | May | 13 | 7 | 20 | 0 | 0 | 0 |
|  | June | 83 | 181 | 264 | 25 | 68 | 93 |
|  | July | 159 | 363 | 522 | 107 | 503 | 610 |
|  | August | 217 | 505 | 722 | 50 | 133 | 183 |
|  | September | 337 | 537 | 874 | 54 | 143 | 197 |
|  | October | 6 | 12 | 18 | 8 | 9 | 17 |
|  | November | 0 | 0 | 0 | 3 | 3 | 6 |
|  | December | 0 | 0 | 0 | 0 | 0 | 0 |
|  | Total | 815 | 1605 | 2420 | 247 | 859 | 1106 |
