# Supplementary material for: Seasonal Dynamics of Phlebotomine Sand Fly Species Proven Vectors of Mediterranean Leishmaniasis Caused by Leishmania infantum
Source: PLoS Negl Trop Dis. 2016 Feb 22;10(2):e0004458. doi: 10.1371/journal.pntd.0004458 (PMC4762948; doi:10.1371/journal.pntd.0004458)
Supplement: S6 Table — (DOCX) [file pntd.0004458.s007.docx]

Table S6. Phlebotomine sand fly species collected in Fodele, Crete, Greece

| Year | Month | *P. similis* | | Total | *S. minuta* | | Total | *P. neglectus* | | Total | *P. papatasi* | | Total |
| --- | --- | --- | --- | --- | --- | --- | --- | --- | --- | --- | --- | --- | --- |
|  |  | Female | Male |  | Female | Male |  | Female | Male |  | Female | Male |  |
| 2011 | April | 0 | 0 | 0 | 0 | 0 | 0 | 0 | 0 | 0 | 0 | 0 | 0 |
|  | May | 0 | 0 | 0 | 0 | 0 | 0 | 0 | 0 | 0 | 0 | 0 | 0 |
|  | June | 56 | 59 | 115 | 0 | 0 | 0 | 6 | 27 | 33 | 0 | 3 | 3 |
|  | July | 102 | 162 | 264 | 13 | 15 | 28 | 6 | 18 | 24 | 0 | 3 | 3 |
|  | August | 15 | 19 | 34 | 8 | 16 | 24 | 55 | 80 | 135 | 1 | 5 | 6 |
|  | September | 11 | 22 | 33 | 10 | 14 | 24 | 22 | 50 | 72 | 2 | 2 | 4 |
|  | October | 1 | 3 | 4 | 2 | 0 | 2 | 1 | 6 | 7 | 0 | 0 | 0 |
|  | November | 0 | 0 | 0 | 0 | 0 | 0 | 0 | 0 | 0 | 0 | 0 | 0 |
|  | Total | 185 | 265 | 450 | 33 | 45 | 78 | 90 | 181 | 271 | 3 | 13 | 16 |
| 2012 | April | 0 | 0 | 0 | 0 | 2 | 2 | 0 | 9 | 9 | 0 | 0 | 0 |
|  | May | 2 | 1 | 3 | 6 | 4 | 10 | 21 | 92 | 113 | 3 | 2 | 5 |
|  | June | 123 | 100 | 223 | 4 | 7 | 11 | 9 | 25 | 34 | 6 | 8 | 14 |
|  | July | 140 | 146 | 286 | 7 | 20 | 27 | 60 | 103 | 163 | 4 | 5 | 9 |
|  | August | 20 | 39 | 59 | 23 | 19 | 42 | 109 | 241 | 350 | 5 | 6 | 11 |
|  | September | 8 | 14 | 22 | 25 | 24 | 49 | 42 | 101 | 143 | 1 | 3 | 4 |
|  | October | 2 | 1 | 3 | 2 | 1 | 3 | 18 | 68 | 86 | 2 | 2 | 4 |
|  | November | 0 | 0 | 0 | 0 | 0 | 0 | 0 | 0 | 0 | 0 | 0 | 0 |
|  | Total | 295 | 301 | 596 | 67 | 77 | 144 | 259 | 639 | 898 | 21 | 26 | 47 |
| 2013 | April | 0 | 0 | 0 | 0 | 2 | 2 | 1 | 6 | 7 | 0 | 0 | 0 |
|  | May | 4 | 9 | 13 | 3 | 5 | 8 | 126 | 294 | 420 | 3 | 4 | 7 |
|  | June | 154 | 148 | 302 | 42 | 20 | 62 | 18 | 40 | 58 | 11 | 13 | 24 |
|  | July | 143 | 144 | 287 | 34 | 48 | 82 | 213 | 277 | 490 | 8 | 8 | 16 |
|  | August | 30 | 38 | 68 | 33 | 41 | 74 | 318 | 420 | 738 | 6 | 8 | 14 |
|  | September | 49 | 40 | 89 | 35 | 19 | 54 | 72 | 222 | 294 | 6 | 7 | 13 |
|  | October | 4 | 4 | 8 | 2 | 5 | 7 | 47 | 105 | 152 | 0 | 0 | 0 |
|  | November | 1 | 1 | 2 | 0 | 0 | 0 | 7 | 18 | 25 | 0 | 0 | 0 |
|  | Total | 385 | 384 | 769 | 149 | 140 | 289 | 802 | 1382 | 2184 | 34 | 40 | 74 |
