# Supplementary material for: Seasonal Dynamics of Phlebotomine Sand Fly Species Proven Vectors of Mediterranean Leishmaniasis Caused by Leishmania infantum
Source: PLoS Negl Trop Dis. 2016 Feb 22;10(2):e0004458. doi: 10.1371/journal.pntd.0004458 (PMC4762948; doi:10.1371/journal.pntd.0004458)
Supplement: S7 Table — (DOCX) [file pntd.0004458.s008.docx]

Table S7. Phlebotomine sand fly species collected in Steni, Cyprus

| Year | Month | *P. neglectus* | | Total | *P. papatasi* | | Total | *P. tobbi* | | Total | *P. galilaeus* | | Total | *P. sergenti* | | *Total* | *Sergentomyia spp* | | Total |
| --- | --- | --- | --- | --- | --- | --- | --- | --- | --- | --- | --- | --- | --- | --- | --- | --- | --- | --- | --- |
|  |  | Female | Male |  | Female | Male |  | Female | Male |  | Female | Male |  | Female | Male |  | Female | Male |  |
| 2012 | April | 0 | 0 | 0 | 0 | 0 | 0 | 0 | 0 | 0 | 0 | 0 | 0 | 0 | 0 | 0 | 0 | 0 | 0 |
|  | May | 0 | 0 | 0 | 1 | 0 | 1 | 4 | 3 | 7 | 1 | 2 | 3 | 0 | 0 | 0 | 2 | 2 | 4 |
|  | June | 0 | 0 | 0 | 3 | 3 | 6 | 3 | 4 | 7 | 0 | 1 | 1 | 0 | 0 | 0 | 26 | 12 | 38 |
|  | July | 0 | 0 | 0 | 1 | 5 | 6 | 15 | 8 | 23 | 0 | 1 | 1 | 0 | 0 | 0 | 34 | 32 | 66 |
|  | August | 0 | 0 | 0 | 81 | 108 | 189 | 38 | 29 | 67 | 8 | 6 | 14 | 0 | 0 | 0 | 43 | 15 | 58 |
|  | September | 0 | 0 | 0 | 78 | 66 | 144 | 68 | 28 | 96 | 15 | 9 | 24 | 0 | 1 | 1 | 12 | 16 | 28 |
|  | October | 0 | 0 | 0 | 20 | 29 | 49 | 10 | 7 | 17 | 0 | 0 | 0 | 0 | 0 | 0 | 3 | 1 | 4 |
|  | November | 0 | 0 | 0 | 0 | 0 | 0 | 0 | 0 | 0 | 0 | 0 | 0 | 0 | 0 | 0 | 0 | 0 | 0 |
|  | Total | 0 | 0 | 0 | 184 | 211 | 395 | 138 | 79 | 217 | 24 | 19 | 43 | 0 | 0 | 0 | 120 | 78 | 198 |
| 2013 | April | 0 | 0 | 0 | 1 | 5 | 6 | 9 | 5 | 14 | 2 | 0 | 2 | 0 | 0 | 0 | 0 | 0 | 0 |
|  | May | 0 | 0 | 0 | 1 | 5 | 6 | 57 | 21 | 78 | 8 | 5 | 13 | 0 | 0 | 0 | 12 | 17 | 29 |
|  | June | 2 | 1 | 3 | 9 | 14 | 23 | 13 | 11 | 24 | 3 | 1 | 4 | 0 | 2 | 2 | 57 | 61 | 118 |
|  | July | 0 | 0 | 0 | 35 | 58 | 93 | 97 | 28 | 125 | 8 | 5 | 13 | 1 | 2 | 3 | 347 | 294 | 641 |
|  | August | 0 | 0 | 0 | 56 | 74 | 130 | 20 | 15 | 35 | 3 | 1 | 4 | 0 | 0 | 0 | 132 | 117 | 249 |
|  | September | 0 | 0 | 0 | 67 | 92 | 159 | 117 | 91 | 208 | 8 | 5 | 13 | 3 | 3 | 6 | 37 | 71 | 108 |
|  | October | 0 | 0 | 0 | 57 | 62 | 119 | 15 | 19 | 34 | 1 | 1 | 2 | 0 | 0 | 0 | 10 | 7 | 17 |
|  | November | 0 | 0 | 0 | 0 | 0 | 0 | 0 | 0 | 0 | 0 | 0 | 0 | 0 | 0 | 0 | 10 | 7 | 17 |
|  | Total | 2 | 1 | 3 | 226 | 310 | 536 | 328 | 190 | 518 | 33 | 18 | 51 | 4 | 8 | 12 | 605 | 574 | 1179 |
