# Supplementary material for: Seasonal Dynamics of Phlebotomine Sand Fly Species Proven Vectors of Mediterranean Leishmaniasis Caused by Leishmania infantum
Source: PLoS Negl Trop Dis. 2016 Feb 22;10(2):e0004458. doi: 10.1371/journal.pntd.0004458 (PMC4762948; doi:10.1371/journal.pntd.0004458)
Supplement: S8 Table — (DOCX) [file pntd.0004458.s009.docx]

Table S8. Phlebotomine sand fly species collected in 6 sites of Cukurova region, Turkey

| Year | Month | *P. papatasi* | | Total | *S. dentata* | | Total | *P. tobbi* | | Total | *P. p. transcaucasicus* | | Total | *P. sergenti* | | Total | *P. major s.l.* | | Total |
| --- | --- | --- | --- | --- | --- | --- | --- | --- | --- | --- | --- | --- | --- | --- | --- | --- | --- | --- | --- |
|  |  | Female | Male |  | Female | Male |  | Female | Male |  | Female | Male |  | Female | Male |  | Female | Male |  |
| 2011 | May | 1 | 5 | 6 | 1 | 4 | 5 | 12 | 121 | 133 | 0 | 0 | 0 | 0 | 0 | 0 | 0 | 4 | 4 |
|  | June | 80 | 55 | 135 | 49 | 9 | 58 | 37 | 636 | 673 | 0 | 24 | 24 | 8 | 6 | 14 | 0 | 17 | 17 |
|  | July | 37 | 10 | 47 | 18 | 10 | 28 | 10 | 91 | 101 | 1 | 23 | 24 | 3 | 2 | 5 | 0 | 0 | 0 |
|  | August | 47 | 28 | 75 | 106 | 54 | 160 | 128 | 382 | 510 | 4 | 132 | 136 | 3 | 12 | 15 | 2 | 14 | 16 |
|  | September | 222 | 139 | 361 | 148 | 86 | 234 | 465 | 1121 | 1586 | 1 | 211 | 212 | 5 | 10 | 15 | 2 | 27 | 29 |
|  | October | 28 | 22 | 50 | 3 | 1 | 4 | 252 | 794 | 1046 | 3 | 279 | 282 | 0 | 0 | 0 | 0 | 16 | 16 |
|  | November | 0 | 0 | 0 | 0 | 0 | 0 | 0 | 0 | 0 | 0 | 0 | 0 | 0 | 0 | 0 | 0 | 0 | 0 |
|  | Total | 415 | 259 | 674 | 325 | 164 | 489 | 904 | 3145 | 4049 | 9 | 652 | 661 | 19 | 30 | 49 | 4 | 68 | 72 |
| 2012 | May | 4 | 2 | 6 | 1 | 0 | 1 | 2 | 136 | 138 | 0 | 1 | 1 | 0 | 0 | 0 | 0 | 1 | 1 |
|  | June | 40 | 61 | 101 | 129 | 54 | 183 | 110 | 357 | 467 | 107 | 383 | 490 | 1 | 3 | 4 | 0 | 2 | 2 |
|  | July | 55 | 48 | 103 | 170 | 178 | 348 | 78 | 361 | 439 | 20 | 149 | 169 | 8 | 3 | 11 | 0 | 2 | 2 |
|  | August | 314 | 217 | 531 | 142 | 22 | 164 | 414 | 1309 | 1723 | 1543 | 4071 | 5614 | 4 | 12 | 16 | 4 | 14 | 18 |
|  | September | 242 | 128 | 370 | 368 | 83 | 451 | 680 | 2222 | 2902 | 1236 | 2549 | 3785 | 9 | 9 | 18 | 5 | 19 | 24 |
|  | October | 53 | 64 | 117 | 72 | 35 | 107 | 398 | 804 | 1202 | 618 | 1757 | 2375 | 1 | 6 | 7 | 3 | 12 | 15 |
|  | November | 0 | 0 | 0 | 0 | 0 | 0 | 0 | 0 | 0 | 0 | 0 | 0 | 0 | 0 | 0 | 0 | 0 | 0 |
|  | Total | 708 | 520 | 1228 | 882 | 372 | 1254 | 1682 | 5189 | 6871 | 3524 | 8910 | 12434 | 23 | 33 | 56 | 12 | 50 | 62 |
