# Supplementary material for: Seasonal Dynamics of Phlebotomine Sand Fly Species Proven Vectors of Mediterranean Leishmaniasis Caused by Leishmania infantum
Source: PLoS Negl Trop Dis. 2016 Feb 22;10(2):e0004458. doi: 10.1371/journal.pntd.0004458 (PMC4762948; doi:10.1371/journal.pntd.0004458)
Supplement: S9 Table — (DOCX) [file pntd.0004458.s010.docx]

Table S9. Phlebotomine sand fly species collected in 2 sites of Tbilisi, Georgia

| Year | Month | *P. sergenti* | | Total | *P. balcanicus* | | Total | *P. halepensis* | | Total | *P. kandelakii* | | Total | *P. wenyoni* | | Total |
| --- | --- | --- | --- | --- | --- | --- | --- | --- | --- | --- | --- | --- | --- | --- | --- | --- |
|  |  | Female | Male |  | Female | Male |  | Female | Male |  | Female | Male |  | Female | Male |  |
| 2011 | April | 0 | 0 | 0 | 0 | 0 | 0 | 0 | 0 | 0 | 0 | 0 | 0 | 0 | 0 | 0 |
|  | May | 0 | 0 | 0 | 0 | 0 | 0 | 0 | 0 | 0 | 0 | 0 | 0 | 0 | 0 | 0 |
|  | June | 0 | 1 | 1 | 0 | 0 | 0 | 0 | 0 | 0 | 0 | 0 | 0 | 0 | 0 | 0 |
|  | July | 34 | 98 | 132 | 12 | 14 | 26 | 5 | 9 | 14 | 1 | 2 | 3 | 3 | 0 | 3 |
|  | August | 73 | 111 | 184 | 6 | 9 | 15 | 3 | 1 | 4 | 0 | 1 | 1 | 0 | 0 | 0 |
|  | September | 17 | 13 | 30 | 1 | 1 | 2 | 0 | 0 | 0 | 0 | 0 | 0 | 0 | 0 | 0 |
|  | October | 0 | 0 | 0 | 0 | 0 | 0 | 0 | 0 | 0 | 0 | 0 | 0 | 0 | 0 | 0 |
|  | Total | 124 | 223 | 347 | 19 | 24 | 43 | 8 | 10 | 18 | 1 | 3 | 4 | 3 | 0 | 3 |
| 2012 | April | 0 | 0 | 0 | 0 | 0 | 0 | 0 | 0 | 0 | 0 | 0 | 0 | 0 | 0 | 0 |
|  | May | 0 | 0 | 0 | 0 | 0 | 0 | 0 | 0 | 0 | 0 | 0 | 0 | 0 | 0 | 0 |
|  | June | 2 | 5 | 7 | 1 | 0 | 1 | 0 | 0 | 0 | 0 | 0 | 0 | 0 | 0 | 0 |
|  | July | 29 | 49 | 78 | 5 | 5 | 10 | 1 | 3 | 4 | 1 | 3 | 4 | 0 | 2 | 2 |
|  | August | 24 | 43 | 67 | 3 | 6 | 9 | 2 | 1 | 3 | 0 | 1 | 1 | 0 | 0 | 0 |
|  | September | 0 | 0 | 0 | 0 | 0 | 0 | 0 | 3 | 3 | 0 | 0 | 0 | 0 | 0 | 0 |
|  | October | 0 | 0 | 0 | 0 | 0 | 0 | 0 | 0 | 0 | 0 | 0 | 0 | 0 | 0 | 0 |
|  | Total | 55 | 97 | 152 | 9 | 11 | 20 | 3 | 7 | 10 | 1 | 4 | 5 | 0 | 2 | 2 |
| 2013 | April | 0 | 0 | 0 | 0 | 0 | 0 | 0 | 0 | 0 | 0 | 0 | 0 | 0 | 0 | 0 |
|  | May | 0 | 0 | 0 | 0 | 0 | 0 | 0 | 0 | 0 | 0 | 0 | 0 | 0 | 0 | 0 |
|  | June | 2 | 5 | 7 | 0 | 0 | 0 | 0 | 0 | 0 | 0 | 4 | 4 | 0 | 0 | 0 |
|  | July | 36 | 85 | 121 | 27 | 17 | 44 | 1 | 3 | 4 | 8 | 21 | 29 | 0 | 0 | 0 |
|  | August | 13 | 38 | 51 | 13 | 13 | 26 | 3 | 5 | 8 | 5 | 13 | 18 | 0 | 1 | 1 |
|  | September | 1 | 2 | 3 | 1 | 1 | 2 | 0 | 0 | 0 | 1 | 1 | 2 | 0 | 0 | 0 |
|  | October | 0 | 0 | 0 | 0 | 0 | 0 | 0 | 0 | 0 | 0 | 0 | 0 | 0 | 0 | 0 |
|  | Total | 52 | 130 | 182 | 41 | 31 | 72 | 4 | 8 | 12 | 14 | 39 | 53 | 0 | 1 | 1 |
