# Supplementary material for: Seasonal Dynamics of Phlebotomine Sand Fly Species Proven Vectors of Mediterranean Leishmaniasis Caused by Leishmania infantum
Source: PLoS Negl Trop Dis. 2016 Feb 22;10(2):e0004458. doi: 10.1371/journal.pntd.0004458 (PMC4762948; doi:10.1371/journal.pntd.0004458)
Supplement: S10 Table — (DOCX) [file pntd.0004458.s011.docx]

Table S10. Results of phlebotomine sand fly collections in sites where miniature CDC light trapping (CDC) was regularly associated with sticky paper trapping (ST)

**Portugal**

| Lisbon+Algarve | *P. ariasi* | | Total | *S. minuta* | | Total | *P. perniciosus* | | Total | *P. sergenti* | | Total | Overtotal | |
| --- | --- | --- | --- | --- | --- | --- | --- | --- | --- | --- | --- | --- | --- | --- |
|  | CDC | ST |  | CDC | ST |  | CDC | ST |  | CDC | ST |  | CDC | ST |
| 2011-2013 | 66 | 40 | 106 | 392 | 2929 | 3321 | 919 | 889 | 1808 | 71 | 70 | 141 | 1448 | 3928 |
| % | 62.3 | 37.7 |  | 11.8 | 88.2 |  | 50.8 | 49.2 |  | 50.4 | 49.6 |  | 26.9 | 73.1 |

**Spain**

| Fuenlabrada | *S. minuta* | | Total | *P. perniciosus* | | Total | *P. papatasi* | | Total | *P. sergenti* | | Total | Overtotal | |
| --- | --- | --- | --- | --- | --- | --- | --- | --- | --- | --- | --- | --- | --- | --- |
|  | CDC | ST |  | CDC | ST |  | CDC | ST |  | CDC | ST |  | CDC | ST |
| 2012-2013 | 754 | 5849 | 6603 | 11515 | 11608 | 23123 | 0 | 1 | 1 | 2 | 0 | 2 | 12271 | 17458 |
| % | 11.4 | 88.6 |  | 49.8 | 50.2 |  | 0 | 100 |  | 100 | 0 |  | 41.3 | 58.7 |

**France**

| Roquedur-le-haut | *P. ariasi* | | Total | *S. minuta* | | Total | *P. perniciosus* | | Total | *P. mascittii* | | Total | Overtotal | |
| --- | --- | --- | --- | --- | --- | --- | --- | --- | --- | --- | --- | --- | --- | --- |
|  | CDC | ST |  | CDC | ST |  | CDC | ST |  | CDC | ST |  | CDC | ST |
| 2011-2013 | 8891 | 5548 | 14439 | 31 | 926 | 957 | 26 | 49 | 75 | 12 | 5 | 17 | 8960 | 6528 |
| % | 61.6 | 38.4 |  | 3.2 | 96.8 |  | 34.7 | 65.3 |  | 70.6 | 29.4 |  | 57.9 | 42.1 |

**Italy**

| Frascati | *P. perniciosus* | | Total | *S. minuta* | | Total | Overtotal | |
| --- | --- | --- | --- | --- | --- | --- | --- | --- |
|  | CDC | ST |  | CDC | ST |  | CDC | ST |
| 2011-2012 | 1192 | 359 | 1551 | 0 | 4898 | 4898 | 1192 | 5257 |
| % | 76.9 | 23.1 |  | 0 | 100 |  | 18.5 | 81.5 |

**Greece**

| Fodele | *P. similis* | | Total | *P. neglectus* | | Total | *P. papatasi* | | Total | Overtotal | |
| --- | --- | --- | --- | --- | --- | --- | --- | --- | --- | --- | --- |
|  | CDC | ST |  | CDC | ST |  | CDC | ST |  | CDC | ST |
| 2011-2013 | 1699 | 116 | 1815 | 3011 | 342 | 3353 | 106 | 31 | 137 | 4816 | 489 |
| % | 93.6 | 6.4 |  | 89.8 | 10.2 |  | 77.4 | 22.6 |  | 90.8 | 9.2 |

**Cyprus**

| Steni | *P. papatasi* | | Total | *P. tobbi* | | Total | *P. galilaeus* | | Total | *P. sergenti* | | Total | Overtotal | |
| --- | --- | --- | --- | --- | --- | --- | --- | --- | --- | --- | --- | --- | --- | --- |
|  | CDC | ST |  | CDC | ST |  | CDC | ST |  | CDC | ST |  | CDC | ST |
| 2012-2013 | 778 | 153 | 931 | 726 | 9 | 735 | 92 | 2 | 94 | 12 | 0 | 12 | 1608 | 164 |
| % | 83.6 | 16.4 |  | 98.8 | 1.2 |  | 97.9 | 2.1 |  | 100 | 0 |  | 90.7 | 9.3 |

**Cumulative data from all sites**

|  | *P. ariasi* | | *P. perniciosus* | | *P. sergenti* | | *P. papatasi* | | *P. neglectus* | | *P. similis* | | *P. tobbi* | | *P. galilaeus* | | *S. minuta* | | *P. mascittii* | | Overtotal | |
| --- | --- | --- | --- | --- | --- | --- | --- | --- | --- | --- | --- | --- | --- | --- | --- | --- | --- | --- | --- | --- | --- | --- |
|  | CDC | ST | CDC | ST | CDC | ST | CDC | ST | CDC | ST | CDC | ST | CDC | ST | CDC | ST | CDC | ST | CDC | ST | CDC | ST |
| 2011-2013 | 8957 | 5588 | 13652 | 12905 | 85 | 70 | 884 | 184 | 3011 | 342 | 1699 | 116 | 726 | 9 | 92 | 2 | 1177 | 14602 | 12 | 5 | 21335 | 27295 |
| Total | 14545 | | 26557 | | 155 | | 1068 | | 3353 | | 1815 | | 735 | | 94 | | 15779 | | 17 | | 48630 | |
| % | 61.6 | 38.4 | 51.4 | 48.6 | 54.8 | 45.2 | 82.8 | 17.2 | 89.8 | 10.2 | 93.6 | 6.4 | 98.8 | 1.2 | 97.9 | 2.1 | 7.5 | 92.5 | 70.6 | 29.4 | 43.9 | 56.1 |
