# Supplementary material for: Seasonal Dynamics of Phlebotomine Sand Fly Species Proven Vectors of Mediterranean Leishmaniasis Caused by Leishmania infantum
Source: PLoS Negl Trop Dis. 2016 Feb 22;10(2):e0004458. doi: 10.1371/journal.pntd.0004458 (PMC4762948; doi:10.1371/journal.pntd.0004458)
Supplement: S11 Table — (PDF) [file pntd.0004458.s012.pdf]

S11 Table. Comparison of temperatures and vector abundances recorded in each site between different years. Examples are given of the interdependency between i) average monthly temperatures registered at the beginning of the sand fly activity and magnitude of peaks; and ii) average summer temperatures and total vector abundance (see Notes).

Notes

i) Average monthly temperatures (°C) were put in correlation with the number of vector specimens collected during a main peak, consisting in a curve described by 3 values. Monthly average temperatures preceding and concomitant with the peak occurrence were considered.

ii) Average temperature of the period from May through October was put in correlation with total vector specimens collected in the season.

P. perniciosus – Algarve, Portugal

<table>
<tr><th>2011</th><th>2013</th></tr>
<tr><td>
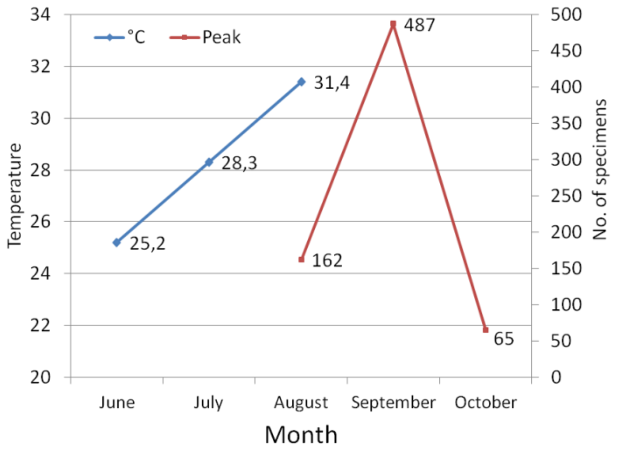

</td><td>
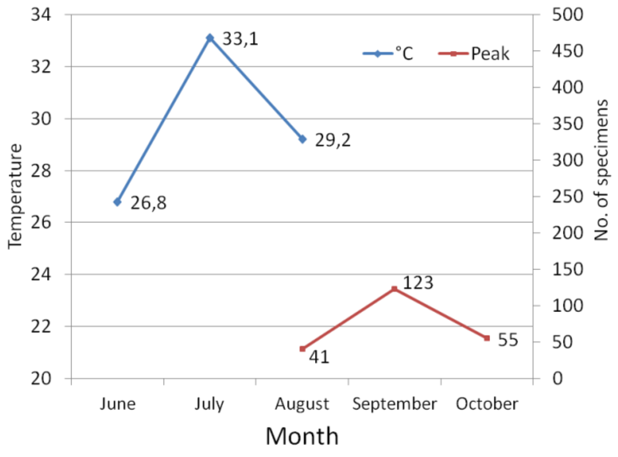

</td></tr>
<tr><td>Average summer temperature: 26.7; Total specimens: 844</td><td>Average summer temperature: 28.2; Total specimens: 354</td></tr>
</table>

P. perniciosus – Spain

2012

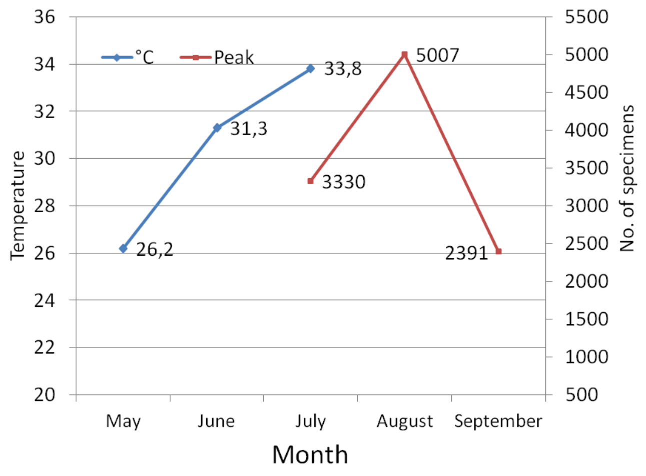

Average summer temperature: 29.0; Total specimens: 14101

2013

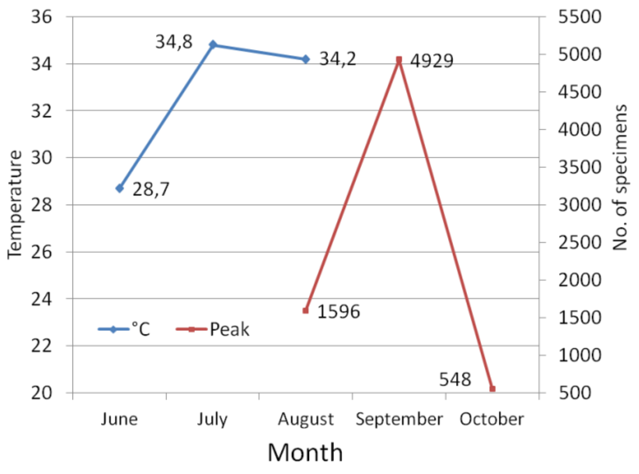

Average summer temperature: 28.3; Total specimens: 9022

P. ariasi – France

2012

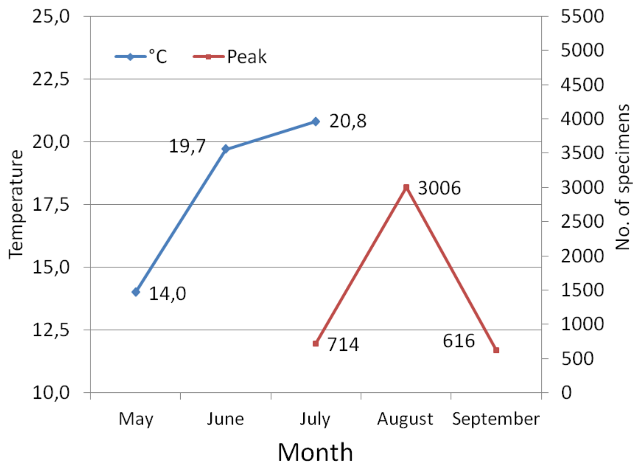

Average summer temperature: 19.5; Total specimens: 4508

2013

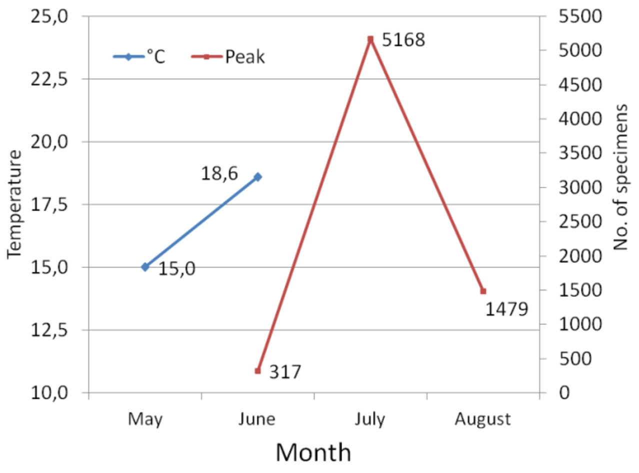

Average summer temperature: 18.8; Total specimens: 7065

P. perniciosus – Italy

2011	2012
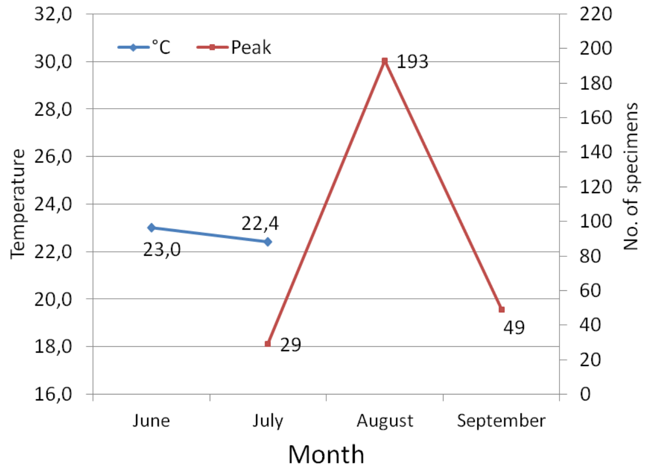	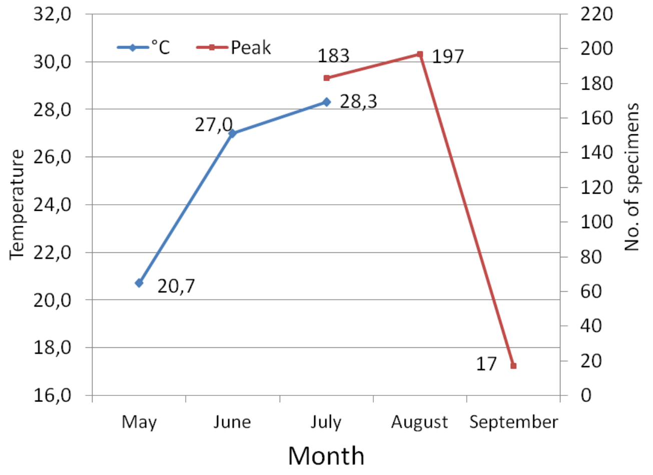
**Average summer temperature: 19.7; Total specimens: 445**	**Average summer temperature: 22.4; Total specimens: 1106**

P. neglectus – Greece

2012	2013
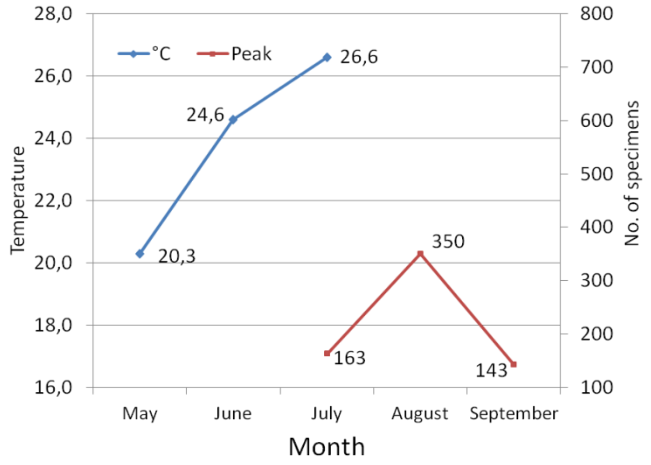	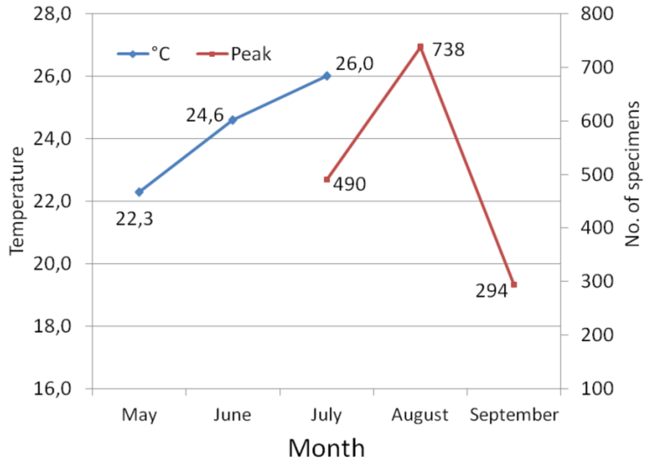
**Average summer temperature: 23.1; Total specimens: 898**	**Average summer temperature: 22.4; Total specimens: 2184**

P. tobbi – Cyprus

2012	2013
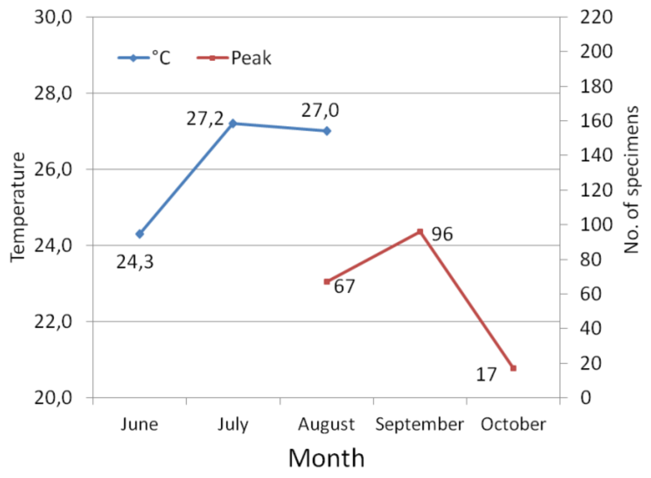	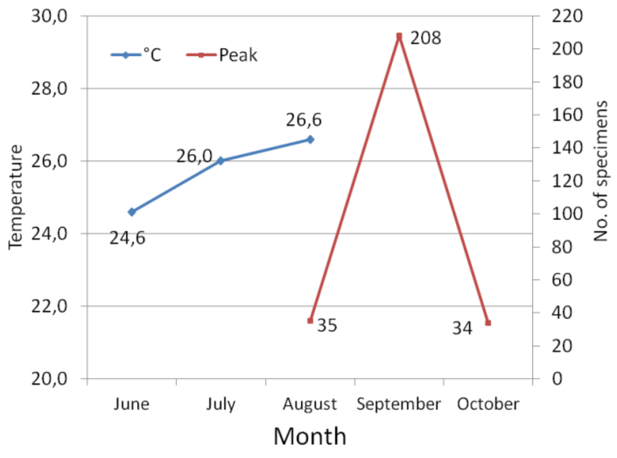

Average summer temperature: 23.9; Total specimens: 217

Average summer temperature: 23.0; Total specimens: 518

P. tobbi – Turkey

2011	2012
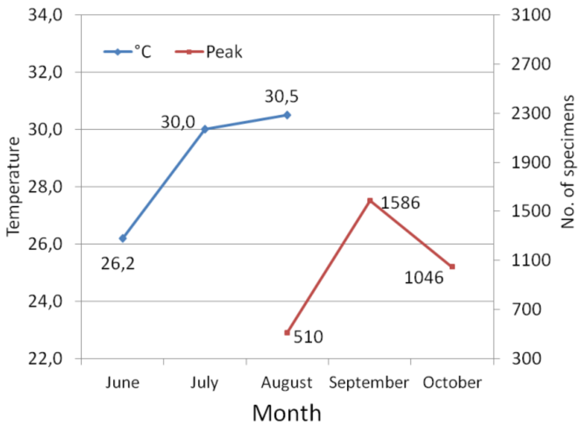	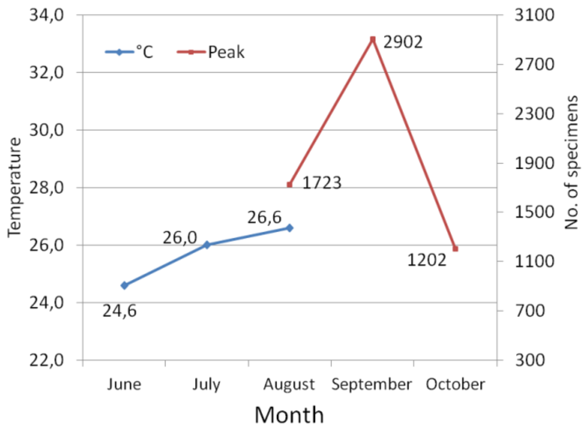

Average summer temperature: 26.9; Total specimens: 4049

Average summer temperature: 27.2; Total specimens: 6871
